# Acknowledgment to the Reviewers of *Nutrients* in 2022

**DOI:** 10.3390/nu15030663

**Published:** 2023-01-28

**Authors:** Nutrients Editorial Office

**Affiliations:** MDPI AG, St. Alban-Anlage 66, 4052 Basel, Switzerland

High-quality academic publishing is built on rigorous peer review. *Nutrients* was able to uphold its high standards for published papers due to the outstanding efforts of our reviewers. Thanks to the efforts of our reviewers in 2022, the median time to first decision was 16 days and the median time to publication was 35 days. Regardless of whether the articles they examined were ultimately published, the editors would like to express their appreciation and thank the following reviewers for the time and dedication that they have shown *Nutrients*:
A. M. HodgeKimon DivarisA. N. M. Mamun-Or-RashidKinan Drak AlsibaiA.K. GroenKinga AdamenkoAaron W. MillerKinga Huminska-lisowskaAarti NagayachKinga MusiałAarti SharmaKinga Stuper-SzablewskaAashima DabasKinga SzczepanekAbbas SmileyKinga TopolskaAbdalla E. El-HadaryKingsley E. AghoAbdelouahed KhalilKinoshita KaoriAbdolreza HosseindoustKirkby D. TickellAbdul RohmanKirsten HerrickAbdulkarim HasanKisuk MinAbdullah Md. SheikhKitamura MineakiAbdullah UsluKiwon LimAbha JainKiyonori KawasakiAbhilasha Kandahalli Venkataranga NayakaKiyotaka UchiyamaAbhinav DubeyKlara KomiciAbhishek ChatterjeeKlaus G. ParhoferAbhishek GuptaKlaus HolzmannAbitha SukumaranKlaus W. LangeAbraham Wall MedranoKnud Erik Bach KnudsenAbrania MarreroKoen Felix Maria JoostenAda ZoharKohei SugiharaAdam DaragóKoichiro MoriAdam HuczyńskiKoichiro TakagiAdamasco CupistiKoji NaruishiAdel PezeshkiKoji SatoAdela BadauKok Hian TanAdele PapettiKolade OluwagbemigunAdil MidaouiKoldo Garcia-EtxebarriaAdina BrahaKomal KalaniAdina ChisKomuraiah MyakalaAdina FrumKonosuke NakajiAdnan Shami ShahKonrad Kamil HozyaszAdolfo Laureano Bautista-CasasnovasKonrad S. JankowskiAdolfo SaiardiKonstantin SharovAdonina TardonKonstantina ArgyriAdrian CãtineanKonstantinos PapadimitriouAdrian CumminsKonstantinos StergiosAdrian FifereKonstantinos TriantafyllouAdrian Florin GalKonstantinos TsioufisAdrian GombartKonstantinos TziomalosAdrian RodriguezKorah Pushpamangalam KuruvillaAdrian Vasile MuresanKossakowska KarolinaAdriana Caldo-SilvaKotha PeddannaAdriana CoppolaKouji MiyazakiAdriana Cristina UrcanKousalya PrabaharAdriana DornellesKowalska MałgorzataAdriana DussoKoya OzawaAdriana FranzeseKozo NakamuraAdriana GrigorașKripa ShankarAdriana Izquierdo LahuertaKris M. MogensenAdriana MikaKrish ChandrasekaranAdriana MonroyKrishna Mohan SurapaneniAdriana R. RodriguesKrishnaji R. KulkarniAdriana VinhasKrishnamoorthy HegdeAdrianne BendichKrista VaradyAdriano MollicaKristen M. RobertsAdrienne ScheckKristijan SkokAfshan MasoodKristin OndrakAftab AhmadKristin StanfordÁgata AranhaKristina SchlichtAgata Białecka-DębekKristina UtzschneiderAgata Carmela NicolosiKristine GriffettAgata ChmurzynskaKristine KrajnakAgata ChudzikKristine UrschelAgata GrzelkaKristóf Endre KárolyAgata MazzagliaKrystian WochnaAgata Poniewierska-BaranKrystyna GutkowskaAgata Sobczyńska-MaleforaKrystyna PawlakAgata StanekKrystyna PawlasAgata WawrzyniakKrystyna RejmanAgata WitczakKrystyna Szymandera-BuszkaAgata ZnamirowskaKrystyna Tyrpień-GolderAgnes TelbiszKrzysztof ChmielowiecAgnieszka Białek-DratwaKrzysztof DołowyAgnieszka Chlebicz-WójcikKrzysztof Gołab˛Agnieszka ChwałczyńskaKrzysztof GorynskiAgnieszka D. Kempińska-PodhorodeckaKrzysztof KaneckiAgnieszka Ewa StępieńKrzysztof LewandowskiAgnieszka HerosimczykKrzysztof SztankeAgnieszka JankowskaKrzysztof SzyfterAgnieszka KamińskaKuan-Han LeeAgnieszka Lipińska-OpałkaKuan-Hsing ChenAgnieszka ŁukomskaKuei-Hui ChanAgnieszka MatuszewskaKuei-I LeeAgnieszka MicekKuen-Chang HsiehAgnieszka NawrockaKumi Nagamoto-CombsAgnieszka OrkuszKumika TomaAgnieszka PawlakKumiko SaekiAgnieszka PiekaraKumpei TokuyamaAgnieszka StawarskaKun LyuAgnieszka Szlagatys-SidorkiewiczKun SongAgnieszka SzmagaraKun YangAgnieszka TudekKunal PalAgnieszka ZabłockaKunal PratapAgnieszka ZagórskaKunihiro SakumaAgostino CasapulloKunka Mohanram RamkumarAgustín HernándezKun-Yi HsinAgustín Llopis-GonzálezKun-Yun YehAhmad AlkhatibKuo-Ching YuanAhmad Al-TitKuo-Chuan HungAhmad Khusairi AzemiKurt A. EscobarAhmed BakillahKushagra BansalAhmed E. Abdel MoneimKwadwo Owusu AkuffoAhmed ElmassryKyle L. ThompsonAhmed R. A. HammamKyle PierceAhmed Ud DinKyle PoulsenAhmed ZahidiKyle TimmermanAhsan HameedKyoji MoritaAhsan JavedKyoko HasebeAida BairamKyriakos KypreosAida TurriniKyu Chang WonAijun QiaoKyung-A HwangAikaterini KonstantinidiKyungho HaAilyn FadriquelaKyung-Jin MinAinhoa Fernández-AtutxaKyung-Wan BaekAirat KayumovKyuwoong KimAirton C. MartinsL. ContrerasAitor LarrañagaL. Manuel-ApolinarAjaikumar B. KunnumakkaraLaavanya RachakondaAjay Kumar MishraLacey McCormackAjay MyneniLadislav ŠtěpánekAjay SharmaLai WeiAjaz AhmadLaisel MartinezAji Arif SabtaLalitha GummidiAkalesh Kumar VermaLamia DahdahAkanksha SharmaLamprou MargaritaAkbar NawabLana NežićAkhilendra Kumar MauryaLanae JoubertAkhtar AliLanfranco D’EliaAkihiko KatoLaPorchia CollinsAkihito TsubotaLara Duran-TrioAkiko SaitoLara Ordóñez-GutiérrezAkiko Sakasai-SakaiLarisa RyskalinAkinari SawadaLarry C. LandsAkinori HaraLars KattnerAkio NakashimaLars Porskjær ChristensenAkio ShimizuLars-Peter KamolzAkio UenoLasse LehtonenAkira IshidaLászló PávicsAkira MinouraLatha RamireddyAkira ObanaLaura Bravo-ClementeAkishige HokugoLaura CianfrugliaAlaa Emara RabeeLaura D’AddezioAlain FreyLaura DeardenAlain LescureLaura Domínguez DíazAlain MassartLaura EspínAlam Md BadrulLaura Giuseppina Di PasquaAlan KellyLaura Grațiela VicașAlan Lins FernandesLaura HopkinsAlanderson Alves RamalhoLaura Inés Elvira-ToralesAlarico ArianiLaura JefferiesAlasdair MacKenzieLaura LessardAlastair ForbesLaura LocatelliAlastair RossLaura Marcos-ZambranoAlba SulajLaura María CompañAlbert F. SmithLaura MasonAlbert KoulmanLaura MitreaAlbert Maimó-BarcelóLaura Muñoz-BermejoAlbertino BigianiLaura OrianAlberto AlfanoLaura ParmaAlberto Enrico MaraoloLaura Perez-Campos MayoralAlberto González-GarcíaLaura PisaniAlberto MiceliLaura Pletsch-BorbaAlberto Rubén OsellaLaura SchulzAlberto SignoreLaura StefaniAlejandra De MorenoLaura Torres-ColladoAlejandra Donají Benítez-ArciniegaLaura WinchesterAlejandra Garcia-AlonsoLaura-María Compañ GabucioAlejandro CarazoLaure PoquetAlejandro Martínez-RodriguezLaurel M. WentzAlejandro Molina LeyvaLauren BrinkAlejandro MuñozLauren DillardAlejandro PlascenciaLauren E. GyllenhammerAlejandro Santos-LozanoLauren HartsteinAlejandro SanzLaurence S. HarbigeAlejandro Sanz-ParisLaurent-Emmanuel MonfouletAleksander J. OwczarekLaurentia Nicoleta GalesAleksander ŚlusarczykLauri ByerleyAleksandra BukhovetsLauri O. ByerleyAleksandra CzumajLaurie Fields DeRoseAleksandra JoticLaurie Nommsen-RiversAleksandra Karczmarska-WódzkaLaurie SvobodaAleksandra KocotLaVerne L. BrownAleksandra KolotaLavinia CasatiAleksandra KołotaLavinia Florina CălinoiuAleksandra KowalskaLavinia PaternosterAleksandra KristoLawrance ChandraAleksandra Nikolić-KokićLawrence SprietAleksandra RymarzLazaros I. SakkasAleksandra S. KristoLe Minh Tu PhanAleksandra SentkowskaLeah M. LipskyAleksandra SzydlowskaLeandro SantiagoAleksandra SzydłowskaLeanne BrownAleksandra Szymczak-TomczakLechosław Pawel ChmielikAleksandra Wisłowska-StanekLechuang ChenAleksandra ZeljkovicLee Samüel WannAlena SalákováLee Wei Rhen WarrenAleš ObrezaLee-Ching HwangAlessandra BordoniLeena KadamAlessandra CamarcaLei WangAlessandra CazzanigaLeigh WardAlessandra ConsalesLeighton JamesAlessandra CosciaLeili Saeednejad ZanjaniAlessandra D’amicoLeis Trabazo María RosauraAlessandra DubbiniLen GoodmanAlessandra ErrigoLene LindbergAlessandra FiorettiLennard SchröderAlessandra GamberucciLeo StevensonAlessandra PullieroLeonard E. GerberAlessandra RivaLeonard StoicaAlessandra RussoLeonardo Catalano IniestaAlessandra SinopoliLeonardo Catalano-IniestaAlessandra ValerioLeonardo De AssisAlessandra ZilliLeonardo Henrique Dalcheco MessiasAlessandro De SireLeonardo Jose Mataruna-Dos-SantosAlessandro DelitalaLeonardo SpatolaAlessandro GranitoLeonid B. LazebnikAlessandro LavianoLeonidas DuntasAlessandro LeoneLeonidas IoannouAlessandro MalobertiLeonie CallawayAlessandro MaugeriLeonie WeinholdAlessandro MengozziLeszek KadzińskiAlessia FilipponeLetian XuAlessia RemiganteLetizia MattiiAlessio AlesciLetizia RasicaAlethia Muñiz-RamirezLetricia Barbosa-PereiraAlex Al KhouryLeyre GravinaAlex Buoite StellaLi CaiAlex Gileles-HillelLi FuAlex MichelsLiana Claudia SalanţăAlex OkaruLiang-Jen WangAlexander BerezinLiang-Tseng KuoAlexander IvanovLiao WangAlexander LarinLiat NachshonAlexander MichelsLibo TanAlexander P. J. HoudijkLidia DaimielAlexander SchaussLidia Hanna MarkiewiczAlexander SerguninLidia SantarpiaAlexander V. OleskinLifeng ZhuAlexander WaitsLigen YuAlexandra AvlonitiLigia DominguezAlexandra CaziucLi-Han ChenAlexandra DiFeliceantonioLi-Han HsuAlexandra FoscolouLihong HaoAlexandra J. MayhewLi-Jen LeeAlexandra-Maria MichaelidouLijuan SunAlexandros PapachristodoulouLijun ZhaoAlexandru BurlacuLila M. OyamaAlexandru Florin RogobeteLilac Lev-AriAlexandru VlaicuLilach SoreqAlexia BarbarossaLili LiAlexios BatrakoulisLili QinAlexis StamatikosLilia AntonovaAlfonso Luca PendolinoLiliana Cristina SoareAlfonso SchiaviLiliana GarneataAlfonso Varela-LópezLiliana KiczakAlfred O. AnkrahLiliana MajkowskaAlfredo CaturanoLilith Caballero AguilarAlfredo Cruz-GregorioLiliya ZiganshinaAlfredo Di LeoLin QiuAlfredo G. CasanovaLin XieAlfredo IrurtiaLin ZhuAli AbbaraLina BegdacheAli BahadurLina GubhajuAli BoolaniLina TingöAli Ehsan SifatLinda AdairAli SaeedLinda Azevedo KauppilaAliakbar AlizadehLinda M. Oude GriepAlice BonomiLinda VeselAlice GiontellaLindsay BrownAlice MonzaniLindsay H. AllenAlice OwenLindsay M. ReynoldsAlicia HeathLindsay N. KohlerAlicja BortkiewiczLindsey BurrellAlicja ChrzanowskaLindsey Haynes-MaslowAlicja Ewa RatajczakLindsy KassAlicja KluczykLine M. OldervollAlicja PonderLine MasséAlida Kindt-DunjkoLine RodeAliki-Eleni FarmakiLingsha JuAlin CiobicaLionel UlmannAlin Mihai VasilescuLipin JiAlina Delia PopaLiping LuAlina DimaLiping SunAlina NegruLisa A. LangsetmoAlina PykaLisa Juntti-BerggrenAline Alves FerreiraLisa LungaroAline AndresLisa RafalsonAline Boveto SantamarinaLisa RubinAline MarcadentiLisa VorkAline Silva Mello CésarLisbet BrandiAlireza ZarebidokiLise AunsholtAlison J. ForheadLisethe MeijerAlison L. EldridgeLisse Angarita DavilaAlison PurvisLi-Tang TsaiAlison SleighLiudmila Gerasimova-MeigalAlison TovarLīva AumeistereAlistair V. W. NunnLivio LuziAllen D. SmithLiwei ChenAllison B. ReissLiya LiAllison HodgeLiyan YangAlmudena González RoviraLjubisa NikolicAlok K. VermaLois JamesÁlvaro Astasio PicadoLola Rueda-RuzafaAlvaro PérezLolita KursvietieneAlyssa HuffLona SandonAlyx TaylorLongxiang SuAmaç Fatih TuyunLongyan ZhaoAmadeus DobrescuLongying ZhaAmadou K.S. CamaraLoredana Liliana HurjuiAmala SoumyanathLoredana RacitiAmali U. AlahakoonLorena DucaAman KumarLorena MardonesAmanda BoydLorena Paola SotoAmanda Cuevas-SierraLorenz S. NeuwirthAmanda GuthLorenza MisturaAmanda LumsdenLorenza TrabalziniAmanda MisselLorenzo GarciaAmanda N. Szabo-ReedLorenzo LippiAmanda V. SardeliLorenzo MalatinoAmanda WallerLorenzo NestiAmany RagabLorenzo Rivas-GarcíaAma-Tawiah EssilfieLorenzo TiduAmber S. KlecknerLorenzo ZanellaAmbrish SinghLoretta R. BrabinAmbrogina AlbergamoLoris PintoAmbrogio P. LonderoLorraine McSweeneyAmel TaibiLothar BreckerAmelia BrunaniLouis Kobina DadzieAmelia CarrLouis Kuoping ChaoAmelia DelgadoLouise A. HorriganAmelia M SilvaLouise BennetAmelia Maria GamanLouise Bjørkholt AndersenAmélie E. CoudertLouise HarveyAmer AhmedLouise JonesAmie SteelLuan Luong ChuAmin N. OlaimatLubomir SkladanyAminde LeopoldLuc BidelAmir ShafatLuca BusettoAmit JaiswalLuca CevolaniAmit KumarLuca CiceroAmitava MukherjeeLuca CimaAmjad KhanLuca Di GianfrancescoAmmad Ahmad FarooqiLuca D’OnofrioAmmar Abdulrahman JairounLuca GallelliAmmar AltemimiLuca Giovanni LocatelloAmmar TahirLuca PaduaAmmu K. RadhakrishnanLuca PangrazziAmna SaharLuca ScalfiAmosy M’KomaLuca SoraciAmr AminLucas D. DiasAmr GamalLucas Opazo-RiosAmra OsmancevicLucia BrodosiAmrendra MishraLucia CarboniAmrik S. KhalsaLucia CarlucciAmrita SarkarLucia CastelliAmutha RamadasLucia F. C. PedrosaAmy Celeste KellerLucia Gimeno-MallenchAmy CunninghamLucia KaiserAmy GaviglioLucia MalaguarneraAmy SuttonLucia Maria Armelin-CorreaAmynah JanmohamedLucía Méndez LópezAna Andabak RoguljLucia MuracaAna B. CerezoLucia StanciakovaAna B. MasedaLucia TerzuoliAna Belén RoperoLucia TonucciAna C. GonçalvesLuciana BaroniAna C. M. G. FigueiredoLuciana Diniz SilvaAna Carolina Silveira RabeloLuciana TorquatiAna CastanhoLucie HasoňováAna Catarina DuarteLucie HénautAna ClaveríaLucie MarousezAna Cordellat MarzalLucija Virovic-JukicAna DascaluLucio CapursoAna DobrevaLucio Della GuardiaAna FariaLucrecia Carrera QuintanarAna Henriques MotaLucyna KozlowskaAna I. Álvarez-MercadoLucyna ScisloAna Isabel Álvarez-MercadoLucyna ŚcisłoAna Isabel EsquifinoLudmila KazdovaAna Lúcia BaltazarLudovica PascaAna Luísa De Sousa-CoelhoLudovico AbenavoliAna M. Cebrián-CuencaLudovico DocimoAna M. López-ParraLuigi CastaldoAna M. PalaciosLuigi Di FilippoAna M. WägnerLuigi GianturcoAna MachadoLuigi GrecoAna María Calderón De La BarcaLuigi MandrichAna Paula Duarte De SouzaLuigi MeccarielloAna Paula FayhLuigi NapolitanoAna PetelinLuigi RosaAna PintoLuigi SantacroceAna Pinto De MouraLuigi SchiavoAna ReisLuigi TarantiniAna RufinoLuigia PazzagliAna TeofilovićLuis Albendín-GarcíaAna Teresa Oliveira RufinoLuis BujandaAna ValadoLuis Cláudio Nascimento Da SilvaAna ValdesLuis Enrique CucaAna ValenciaLuis F. MartinezAnallely López-YerenaLuis GoyaAna-Marija DomijanLuis Ignacio Fernández-SalazarAnand DesaiLuis M. Lou-ArnalAnand ParamasivamLuis Mariano EstebanAnand ThirupathiLuís MartinsAnannya BhattacharyaLuis Miguel EsparzaAnanta Prasad ArukhaLuis Omar Colombo-MendozaAnapaula Sommer VinagreLuis Pereira-da-SilvaAnas M. Abdel RahmanLuís Pereira-da-SilvaAnastasia MarkakiLuis RodrigoAnastasia ThanopoulouLuisa GorzaAnastasiia PetushkovaLuisa LampignanoAnastasios TheodorouLuisella CianferottiAnastazia KeiLuisella VignaAnat Yaskolka MeirLuiz Antonio Del CiampoAnbarasu KannanLuiz Augusto PerandiniAnca Corina FarcasLuiz Fernando Romanholo FerreiraAnca DinischiotuLukas ParkerAnca Lucau-DanilaLukas PfeiferAnca Oana DoceaLukas StalpersAnca PopŁukasz A. PoniatowskiAnca ZguraŁukasz BobakAnchuelo Arturo CorbatónLukasz DembinskiAndaleb KholmukhamedovŁukasz DembińskiAnders LarssonŁukasz DobrekAnderson Oliveira SouzaŁukasz KrystAndor H. MolnárŁukasz OleksyAndra PiciuŁukasz SkoczylasAndras BikovŁukasz SzymañskiAndrás FehérŁukasz TomczykAndras HajnalLuke Kang Kwong KapathyAndraz DovnikLum Peng LimAndré L.L. BachiLuyu XieAndre RodackiLydia ÁlvarezAndré RosárioLydia DevenneyAndre SilvaLynn A. MegeneyAndre WegnerLynn M. PezzaniteAndrea BotturiLynn UlatowskiAndrea BuchholzLyudmila Bel’skayaAndrea CaporaleLyudmila I RachekAndrea CioffiLyudmila KorostovtsevaAndrea Da PortoLyudmila RachekAndrea D’AmatoLyvia BiagiAndrea David Re CecconiM Dolores Ruiz-LópezAndrea DeleddaM Jashim UddinAndrea FinM. Di StefanoAndrea GalosiM. Dulce EstêvãoAndrea GarberM. Kathleen ClarkAndrea Giuseppe MaugeriM. Pilar Fernández-MateosAndrea GodinoM. Purificacion Gonzalez-GonzalezAndrea J. LobeneM. VaqueroAndrea Kopp LugliM. Yanina PepinoAndrea MarchiniM.C. GuerreraAndrea MontagnaniM.Z. JankovicAndrea MonteagudoMaaike KockxAndrea Paola Rojas GilMaarouf AdilAndrea PastaMaarten C. BoslandAndrea PiccinMabel BladesAndrea PoliMaciej GawęckiAndrea RagusaMaciej HałasaAndrea RomaniMaciej KaczmarskiAndrea SambriMaciej KwiatekAndrea SantarelliMaciej PolakAndrea TinelliMaciej SałagaAndrea TuraMaciej W. SochaAndrea VaniaMack ShelleyAndreadi AikateriniMădălin-Iancu EnacheAndreas DoetschMaddalena FratelliAndreas IhleMadelene EricssonAndreas SchulzeMadison L. KackleyAndreas VychytilMagadi SrivathsaAndree Delahaye-DuriezMagda H. AbdellattifAndreea Lili BărbulescuMagda MocanuAndrei BorșaMagdalena Buszewska-ForajtaAndreia CostaMagdalena Czlapka-MatyasikAndrés GiovambattistaMagdalena DudekAndrés Godoy-CumillafMagdalena GórnickaAndrés MorenoMagdalena JankowskaAndrew CollettMagdalena KolasaAndrew CovelerMagdalena Kurnik-ŁuckaAndrew D. FrugéMagdalena Makarewicz-WujecAndrew DasMagdalena Martínez-CañameroAndrew FoskettMagdalena MititeluAndrew FryMagdalena OgłuszkaAndrew HillMagdalena Olszanecka-GlinianowiczAndrew HolwerdaMagdalena Orczyk-PawiłowiczAndrew LavenderMagdalena PaczkowskaAndrew Ming-Liang OngMagdalena Paulina KasprzakAndrew OzgaMagdalena PodbielskaAndrew S. DayMagdalena SkotnickaAndrew SinclairMagdalena Śmiglak-KrajewskaAndrew SpringMagdalena SobieskaAndrew StadnykMagdalena StaszczakAndrew SzilagyiMagdalena SzopaAndrew YenMagdalena Wójciak-KosiorAndrey A. ZamyatninMagdalena ŻegleńAndrey S. LopatinMagdeldin ElgizouliAndriana C. KalioraMagnus SjogrenAndriati NingrumMagnus SjögrenAndrzej BozekMaha HoteitAndrzej CendrowskiMaharshi BhaswantAndrzej CieslikMahendran SekarAndrzej DeptałaMahesh KasthuriAndrzej KoniecznyMahesh Raj NepalAndrzej P. OkoMahmoud Abo-AshourAndrzej PawlikMahmoud AbughoushAndrzej SlominskiMahmoud AbulmeatyAndrzej StawarskiMahmoud BalbaaAndrzej TomczakMahrous Abdelbasset IbrahimAndrzej TukiendorfMahshid NaghashpourAndrzej ZachwiejaMai MatsumotoAndy Po-Yi TsaiMaija Huttunen-LenzAndy WullaertMailo VirtoAndy Yen-Tung TengMaitreyi RamanAneeza W. HamizanMaja BaretićAnella SavianoMaja KopczyńskaAneta BrzezickaMaja MiskulinAneta Cymbaluk-PłoskaMaja ŠantakAneta KopećMaja Šikić PogačarAngel Luis García-VillalónMajed AbuKhaderAngela A. MulliganMajid KoozehchianAngela FraserMajid Mufaqam Syed-AbdulÁngela García-GonzálezMakenzie BarrAngela GuardaMakoto HirakawaAngela GuarinaMakoto NishizukaAngela K. LawsonMakoto ShimizuAngela M. Thompson-PaulMaksim RebezovAngela MeadowsMalbert Charles-HenriAngela PolitoMalcolm RileyAngela RizzoMałgorzata BalaAngela SorrentinoMałgorzata BiałekAngela Tellez-LanceMalgorzata BurekAngela Yen MooreMałgorzata Gietka-CzernelAngela-Patricia HernándezMalgorzata GrembeckaÁngeles Espinosa-CuevasMałgorzata GrzesiukAngeles F. EstévezMałgorzata JamkaAngelia Maleah HollandMałgorzata Kosicka-GębskaAngelia Maleah Holland-WinklerMalgorzata KosteckaAngelica Quintero-FlorezMałgorzata Kotula-BalakAngélica Saraí Jiménez-OsorioMałgorzata KujawskaAngeliki AngelidiMałgorzata LenartowiczAngeliki KatsafadouMałgorzata LewandowskaAngeline ChatelanMałgorzata Małodobra-MazurAngelo BañaresMalgorzata Maria JerzakAngelo M. CarellaMalgorzata MatusiewiczAngelo SabagMalgorzata Miazga-KarskaAngelo TremblayMałgorzata MoczkowskaAngelos SikalidisMałgorzata MoszakAnibh M. DasMałgorzata SztubeckaAnil K. SharmaMałgorzata TabaszewskaAnil K. VermaMalgorzata Tyszka-CzocharaAnil SinghMalgorzata Witkowska-ZimnyAnil VermaMałgorzata WojciechowskaAnindya Sundar RayMałgorzata WroniakAnita MatićMałgorzata WrzosekAnita MikolajczykMalgorzata Zakłos-SzydaAnita Rogowicz-FrontczakMałgorzata ZiarnoAnita Silvana Ilak PeršurićMalin BarmanAnita SubramanianMallikharjuna KoriviAnitra CarrMamdouh SamyAnja Kathrin JaehneMan LiuAnju R. BabuManan RoyAnke WeissenbornMandy StahreAnkie Tan CheungManendra Babu LankadasariAnkit ManglaManickam AshokkumarAnkit ShroffManinder K. AhluwaliaAnkita PunethaManira MaarofAnkur NaqibManisha NaithaniAnn TariniManja M. ZecAnn WebbManju M. ChandraAnna Arnal-GómezManohar KodavatiAnna BarileManoj KumarAnna BartosiewiczMansour A. MahmoudAnna Berthold-PlutaManuel A. MaliqueoAnna BieniekManuel Alonso Olivares GrohnertAnna BiernasiukManuel CoimbraAnna Bilska-WilkoszManuel Durán-PovedaAnna BirukovManuel Gómez-GuzmánAnna BrancatoManuel Pabón-CarrascoAnna BrzeckaManuel PestanaAnna CapozziManuel Ramírez SánchezAnna CasselbrantManuel Sanchez SantosAnna ChmielewskaManuel SorianoAnna CieślińskaManuel Vázquez-CarreraAnna Cleta CroceManuel Viuda-MartosAnna CzubaszekManuela Expósito-RuizAnna DanielewiczManuela MeirelesAnna Di SalleMao-Meng TiaoAnna DrabczykMapuana C. K. AntonioAnna Duda-SobczakMara CarsoteAnna ElkinaMarc BonnefoyAnna Erkiert-PolgujMarc MarescaAnna ErmundMarc P. McRaeAnna FlorowskaMarc T. FacciottiAnna GalickaMarcel NaikAnna GavrieliMarcelina ParrizasAnna GrygierMarcelino Pérez-BermejoAnna HartonMarcello CeciAnna J. MilewskaMarcelo Paes BarrosAnna Jeḑrusek-GolinśkaMarcia Galván-PortilloAnna Kablak-ZiembickaMarcia HiriartAnna KałużaMarcin ArciszewskiAnna KårlundMarcin DziekiewiczAnna KleczkaMarcin GierachAnna Kostecka-GugalaMarcin KosmalskiAnna Luiza Facchetti Vinhaes AssumpçãoMarcin RóżewiczAnna M. Różańska-WalędziakMarcin SchmidtAnna Maria GiustiMarcin SochalAnna Maria NuzzoMarcio Alvarez-SilvaAnna Maria StabileMarco Alexandre Da Silva BatistaAnna Maria TartaglioneMarco Antonio Hernández-LepeAnna MatuszewskaMarco AtteritanoAnna Merwid-La̧dMarco Bauzá ThorbrüggeAnna MichnoMarco BertiniAnna NegroniMarco BiagiAnna Nowak-WegrzynMarco Bruno Luigi RocchiAnna OgrodowczykMarco CarmignaniAnna OlczakMarco CentanniAnna OliverasMarco DacremaAnna OniszczukMarco GiussaniAnna Paradowska-StolarzMarco MalagutiAnna PartykaMarco Matteo CicconeAnna Pérez-BosqueMarco PalumboAnna PerriMarco RinaudoAnna Rafało-UlińskaMarco RiosAnna Romaszko-WojtowiczMarco SegattoAnna RonowskaMarco ValvanoAnna Rzepecka-StojkoMarcos Pereira-SantosAnna SpagnolettaMarcus ElamAnna Stanislawska-SachadynMardelle AtkinsAnna StasiakMaree G. ThorpeAnna StefanskaMarek BrennensthulAnna Szaflarska-PopławskaMarek ŠebelaAnna Tankiewicz-KwedloMarek SkrzypskiAnna ValenzanoMarek VeckaAnna Vila-MartíMargaret MorrisAnna VillariniMargaret OttavianoAnna Vittoria MattioliMargaret SlavinAnna WinkvistMargaret StuberAnna WojciechowskaMargarida Castell EscuerAnna WorthmannMargarida F. B. SilvaAnna WuMargarida VieiraAnna Zalewska-JanowskaMargarita AguileraAnnabel S. Müller-StierlinMargarita TenopoulouAnnalisa BarbieriMargherita CantornaAnnalisa BlasettiMargherita EufemiAnnalisa ChiavaroliMaria A. HorstAnnalisa De SilvestriMaria Aderuza HorstAnnalisa MaiettiMaria Angeles Caballero-MoraAnnalisa NoceMaria Angels CalvoAnnamaria ManciniMaría Antonia Parra-RizoAnnamária PallagMaria Antonietta PanaroAnnamaria ZsakaiMaria ArnedoAnnchen MielmannMaria AzradAnne BlaisMaria BaltogianniAnne DalyMaria BenllochAnne E. BarrettMaria BogdanAnne Marie SowerbuttsMaria Borszewska-KornackaAnne VirsolvyMaria Carla CraveroAnne-Laure Sutter-DallayMaría CarvajalAnnette BrandtMaria Consolata MilettaAnnette StafleuMaria Cristina BarbalaceAnnibale PucaMaria Cristina CaroleoAnnie Hardison-MoodyMaria Cristina CuriaAnnika JansonMaria Cristina GauzziAnn-Kathrin LedererMaria Cristina VerdenelliAnoop KumarMaria Da Graça Costa MiguelAns EilanderMaria Daniel Vaz De AlmeidaAnselm JordaMaría De Jesús Perea FloresAnte PrkićMaria De La Luz Garcia-HernandezAnthia MatsakidouMaría De Los Ángeles Ortega De La TorreAnthony LynnMaria Del Carmen Lozano EstevanAnthony P. JamesMaría Del Mar ContrerasAnthony SclafaniMaría Del Pilar Montero LópezAntje TannenMaria Del Rosario Rico FerreiraAnton KiselevMaria DemetriouAntonela MatanaMaria Do Céu CostaAntonella AgodiMaría Dolores Marrodán SerranoAntonella CaccamoMaría Dolores Ortiz-MasiáAntonella Di SottoMaria Dolores Rivero-PérezAntonella IzzoMaría Dolores Sánchez FernándezAntonella TramutolaMaria Dyah KurniasariAntoni NiedzielskiMaria E. StefanidouAntonia CianciulliMaria Eduardo-FigueiraAntonia García FernándezMaría Elena Acosta-EnriquezAntonia KaltsatouMaria Elena CavicchioloAntonia KondouMaria Elena Vila-SuárezAntonietta RobinoMaría Elizabeth TejeroAntonija TadinMaría Elvira López-OlivaAntonio BarbatoMaría Emilia SantillánAntonio BellastellaMaria Enrica BettinelliAntónio CaleiroMaría Eugenia Jaramillo FloresAntonio CicchellaMaria Felicia SoluriAntonio ColantuoniMaría Fernanda Bernal-OrozcoAntonio CorselloMaria GacekAntonio De Jesús Cenobio-GalindoMaria GrammatikopoulouAntonio E. PontiroliMaria GrauAntonio G. OliveiraMaria Grazia MorgeseAntonio GidaroMaria Grazia SignorelloAntonio GuaitaMaria Grazia TarsitanoAntonio Jesús Martínez-OrtegaMaria Immacolata SpagnuoloAntonio Jr LanchaMaria Irene PreteAntonio LeoMaria Izquierdo PulidoAntonio Miguel Linares-LujánMaría J. PeralAntonio MolinaroMaría Jesús Rodríguez-YoldiAntonio Peña-FernándezMaria João MartinsAntonio Pérez-GálvezMaria João MenesesAntonio PicarelliMaría José Castro-AlijaAntonio PortolesMaría José Muñoz-AlférezAntonios E. KoutelidakisMaría José Soto MéndezAntonios K. GounarisMária KovalskáAntti MeroMaria LinderAnuja PatilMaria Lorella GianniAnujit SarkarMaría Luisa Zagalaz SánchezAnusha DhanasiriMaria Luisa ZeddeAnusha PenumartiMaría M. Morales Suárez-VarelaAnvesh Reddy DasariMaria M. OrbetzovaAnying SongMaria MaaresApostolos PapachristosMaria MaistoApril J. StullMaria MarakiAra KoMaria MarinescuArchana SaxenaMaría MeloArely Vergara-CastañedaMaria MilettaAreta Magda CzerwinskaMaria MirabelliArgha MondalMaria MonsalveArick WangMaria Morgan-BathkeArielle DeutschMaria NotarnicolaArif Sabta AjiMaría Orosia Lucha-LópezArijit NathMaria P. BonasoniAris SiafarikasMaria P.A. BaldassarreAristea GioxariMaria PalmeriniArjan VissinkMaria Paola BertuccioArkadiusz KrzyżanowskiMaria ParpinelArkadiusz LubasMaria Pina ConcasArkadiusz MatwijczukMaria Pina MollicaArkadiusz SzpicerMaria Pokorska-ŚpiewakArland HotchkissMaria Raquel Marçal NataliArleta DrozdMaria Raquel Ventura LucasArlette Suzy SetiawanMaria RicciardiArmando A. Rayas-AmorMaría Roca LlorensArmando Tovar-PalacioMaria RomoArmandodoriano BiancoMaria RussoArmin AlaediniMaria Salud Abellan-RuizArmin ZittermannMaria SarapultsevaArmond DaciMaria SkokouArmond S. GoldmanMaria Sole FacioniArno Schmidt-TrucksässMaria Soto-GironArnulfo Hernan Nava-ZavalaMaria Stefania SpagnuoloArnulfo Ramos-JiménezMaria Teresa Ferreira MoreiraÁron TörökMaria Teresa GuagnanoArpita BasuMaría Teresa Iglesias LópezArrigo CiceroMaria Teresa InzitariArsenio PaezMaria Teresa Nestares PleguezueloArshad PadhiarMaria Teresa PaganoArshi KhanamMaria Teresa PretelArthur GriderMaria Teresa TomasArthur P. WunderlichMaria VenihakiArtur GłuchowskiMaria WitteArtur J. MartinsMaria Zelinda RomanoArtur Jorge Araújo Magalhães RibeiroMariacristina SiottoArtur PałaszMaría-Isabel OrtegaArtur RebeloMarialaura BonaccioArtur SzwengielMarian ButuArturo LópezMarian L. KruzelArulkumar NagappanMariana Buranelo EgeaArun RajasekaranMariana Cecilia CalleArunkumar RanganathanMariana FloriaArushi Gahlot SainiMariane Bittencourt FagundesArwa MustafaMariangela CentroneArzu UluMarianna CarbonneAsad NawazMarianna E WeenerAsaf TzachorMarianna KapetanouAsel RyskulovaMarianna KarachaliouAsher ShafrirMarianna RaczykAshfaq AhmadMarianna RoselliAshleigh HomerMarianna SadagurskiAshley HerdaMariano Francesco CaratozzoloAshley K. PutmanMariantonietta SucciAshok IyaswamyMariapaola MarinoAshok K. ShakyaMariarosaria ValenteAshok K. SinghMaría-Teresa García-ConesaAshot SargsyanMarica BakovicAshraf Al-brakatiMarie MigaudAshutosh BahugunaMarie SjögrenAshutosh PandeyMarie-Claude PaquetteAshwini Namasivayam-MacDonaldMarie-Eve LabonteAsif KhaliqMarie-Laure FrelutAsima ShahMarie-Pierre TalovacciAsmaa KhafagaMarija M. BožićAsnat RazielMarija TakicAsrar AhmadMarijana BasicAsta ByeMarijana SokolovicAstrid HammerMarijana Zovko KončićAstrid Ruiz-MargainMarika Dello RussoAstrida VelenaMarika LanzaAsuka IshiharaMarika MassaroAswathy VijayakumarMariko HosozawaAthanasia PapazafiropoulouMariko MiyazakiAthanasia PrintzaMarilena AntunesAthanasios K AnagnostopoulosMarilena VitaleAthanasios N. TsartsalisMarilia BarrecaAthanasios PapakyriakouMarilia CarabottiAtish MukherjiMarília CravoAtso RaasmajaMariluz Luz Latorre-MoratallaAtsushi HosuiMarilyn CornelisAtsushi IshiharaMarina Aleksandrovna DarenskayaAtsushi IwaiMarina FilimonovaAtsushi KittakaMarina Kiyomi ItoAtsushi SuzukiMarina M. S. Cabral-PintoAtsushi YamashitaMarina QuartuAtte Von WrightMarina SantaellaAttila KissMarina Valente-BarrosoAttila TordaiMarine CouéAttinà AldaMarinka Mravak StipetićAudrey TierneyMarino VilovićAudrius DulskasMario AbateAugustin M. OfiteruMario Albert Ruiz-LópezAura GabrielMario AmatoAurélie GouxMario Bernardo-FilhoAurelijus BurokasMario MiniatiAurelio Lo BuglioMariola JaniszewskaAurelio Luna MaldonadoMariola KozłowskaAureliusz KosendiakMarion HetheringtonAurup Ratan DharMarion RégnierAušra MongirdienėMarion RyanAustin C. HogwoodMarios SpanakisAustin GraybealMarisa GuillénAutumn MarshallMariska Dötsch-KlerkAvik DuttaMaristella GussoniAvinash GothwalMarius BaranauskasAviva MusicusMarius Emil RusuAvraham ShotanMarius MogaAxel HaverichMariusz Aleksander BromkeAya FujiwaraMariusz DabrowskiAyat F. HashimMariusz DąbrowskiAydin Bordbar-KhiabaniMariusz GujskiAyman MahmoudMariusz NiemczykAyoub KasratiMariusz WitczakAysegul AksanMarjo Je Campmans-KuijpersAysegül AksanMark B. MeyerB. Timothy WalshMark BerryBabak PakbinMark C. ChappellBabita BishtMark E. M. ObrenovichBahar BojlulMark Elisabeth Theodorus WillemsBahez GarebMark FarrarBala S. C. KoritalaMark ManaryBalakrishna KoneruMark R.CorkinsBalamurugan RamatchandirinMark TarnopolskyBanafshe HosseiniMarko GerićBao-Peng LiuMarko KumrićBárbara AngelMarko NovakovićBarbara AzzimontiMarko SamardžijaBarbara BlackwellMarko TarleBarbara BorczakMarkos KlonizakisBarbara BuffoliMarkus A. KellerBarbara CuomoMarkus HerrmannBarbara De FilippisMarkus S. JördensBarbara DziedzicMarkus SchäferBarbara FraczekMarkus StraußBarbara GordonMarkus TschurtschenthalerBarbara JachimskaMarkus WilhelmiBarbara Kutryb-Zaja̧cMarlene WewalkaBarbara LiederMarlies Hörmann-WallnerBarbara MavroidiMarloes DekkerBarbara MulloyMarques OrianaBarbara Nieradko-IwanickaMarquez-Rocha Facundo-JoaquinBarbara NikolaidouMarta Arroyo IzagaBarbara Obermayer-PietschMarta BalaskoBarbara OlendzkiMarta Elena Losa IglesiasBarbara Perez VogtMarta GaburjákováBarbara PietruszkaMarta GalloBarbara ReynesMarta HabanovaBarbara Ruszkowska-CiastekMarta HribalBárbara S. RochaMarta Liszka-SkoczylasBarbara SotteroMarta MenegoliBarbara SozanskaMarta MuszalikBarbara SozańskaMarta PelczyńskaBarbara TiozzoMarta PlichtaBarbara Vad AndersenMarta Ramon-KrauelBarbara VeselkaMarta RossiBarbara WięckowskaMarta RusekBarbarella De Matos MacchiMarta SajdakowskaBarry ShaneMarta Swatko-OssorBart De GeestMarta Szandruk-BenderBartłomiej GmajMarta TomczykBartosz KruszewskiMarta Tomczyńska-MlekoBartosz MiziołekMarta WłodarczykBaruh PolisMarta WoźniakBayu Satria WiratamaMartha S. FieldBeata DruzynskaMarthandam Asokan ShibuBeata DrużyńskaMartijn J.J. FinkenBeata HornikMartin Frederik LaursenBeata JabłońskaMartin KeppelBeata ŁoniewskaMartin PecBeata M. Gruber–BzuraMartin RootBeata NarożnaMartin S. LipskyBeata NowakMartin SchepelmannBeata PioreckaMartin SeayBeata Sarecka-HujarMartin SvobodaBeata SperkowskaMartin ValachovicBeata SzluzMartin YeomansBeatrice ArosioMartina BarchittaBéatrice Brembilla-PerrotMartina BednářováBeatrice FemminellaMartina BituhBeatrice Gabriela IoanMartina CaramentiBeatrice ScazzocchioMartina GažarováBeatriz I. Vázquez BeldaMartina Srajer GajdosikBeatriz PereiraMartina ValachovičováBeatriz SarriaMartine ArmandBee Kang TanMartis GeorgianaBeheshteh OlangMarwan E. MajzoubBehnam VafadariMary Beth ArensbergBelem BarbosaMary BoardBelinda López-GalánMary Dawn KoenigBelur R. LokeshMary E. PhillipsBen C. L. ChanMary HicksonBence RaczMary Katherine HoyBenedikt MerzMary M. FlynnBenevides Pessela JoãoMary McKennaBeniamin Oskar GrabarekMary O’connell MotherwayBenjamim FicialMary Rose SweeneyBenjamin A. LernerMary YanBenjamin J. RyanMary YannakouliaBenjamin KochMaryam BoshtamBenjamin WoolfMaryam KebbeBenno WeigmannMaryam Mobed-MiremadiBenoît MarsauxMaryam YuhasBerend IsermannMary-Anne LandBerenice PalaciosMary-Elizabeth PattiBernadette P. MarriottMarzena DominiakBernard LeungMarzena Jeżewska-ZychowiczBernard PossidenteMarzena TomaszewskaBernhard HessMarzena Włodarczyk-StasiakBernhard ReschMarzena WójcikBerta SchnettlerMarzia VasarriBertrand LiagreMaša PintaričBeth MallardMasaaki InabaBetty Yuen-Kwan LawMasaharu KagawaBhagavathi SundaramMasahiro AkiyamaBhagavathi Sundaram SivamaruthiMasahiro YoshikawaBhawik Kumar JainMasakazu SugishimaBhoomika MathurMasako ShimadaBhopal MohapatraMasako ShomuraBhupendra PrajapatiMasamitsu HyodoBhupinder KapoorMasamitsu IchihashiBiagio BaroneMasanori KatakuraBiagio PalmisanoMasao YamasakiBianca Maria TihăuanMasashi FujinoBianca N. JacksonMasashi InafukuBibek PoudelMasataka ShirakiBidisha RoyMasato YasuiBijo MathewMasato YonedaBiju BalakrishnanMasayuki OkudaBilal Alashkar AlhamweMasoumeh Kourosh AramiBimal GhimireMassimiliano EspositoBingde ZhengMassimo CirilloBingyun SunMassimo D’ArchivioBin-Huei ChenMassimo Dal MonteBinxing LiMassimo D’ArchivioBirgitta StrandvikMassimo FaustiniBishnubrata PatraMassimo GrilliBjörn SundströmMassimo LucariniBjörn TampeMassimo MapelliBlaine RobertsMatan ShelomiBlanca Hernández-LedesmaMatas JakubauskasBlanca Riquelme GallegoMatea Zajc PetranovićBlanca Riquelme-GallegoMateja SiršeBlanca Salinas-RocaMateusz GrajekBlanca Sánchez-RamírezMateusz ŁucBlanco-Canosa Juan BautistaMatheus PereiraBlandine LaferrereMathias PossnerBlazen MarijicMathieu MaltaisBlaženka KosMathilde Body-MalapelBlessing Akombi-InyangMatias Monsalves-AlvarezBlessing J. AkombiMatilda Steiner-AsieduBo LiMatilla-Escalante Diana C.Boban MelovćMatsuda KazunoriBo-Chul ShinMatteo Cotta RamusinoBogdan Ionel TambaMatteo Della PortaBogdan TiguMatteo Luigi Giuseppe LeoniBogdana NasuiMatteo MartinatoBogdan-Mircea MihaiMatteo RipaBogna Grygiel-GórniakMatthew A. BaileyBogumiła SzponarMatthew B. McSweeneyBojan KnapMatthew CookBojana ŠarićMatthew Francis MuldoonBojana VidovicMatthew G. BrewerBoldeanu Mihail VirgilMatthew GreeneBolesław KalickiMatthew J. LandryBoleslaw KarwowskiMatthew LittleBonggi LeeMatthew P. KrauseBono LučićMatthew P. LongneckerBoris AntunovićMatthew StrubBoris C Rodríguez-MartínMatthew. J. BarnesBoris De RuyterMatthias KapischkeBoris L. ZybailovMatthias KarstBor-Ran LiMatthias LampingBoyan GrigorovMatthias ZeilerBoyong KimMatti JauhiainenBo-young HongMattia CappellettiBožena CukrowskaMattia Di NunzioBožena Ćurko-CofekMattias CarlstromBożena Szyguła-JurkiewiczMattias PaulssonBożena Targońska-StępniakMaulin PatelBożena TyliszczakMaura E. WalkerBradley R. SalonenMaureen BernerBrandon RestrepoMaureen EvansBrandy-Joe MillironMaureen GroerBrenda SmithMaurizio BalestrinoBret LuickMaurizio Giuseppe AbrignaniBret M. RustMauro Batista De MoraisBrett R. ElyMauro CeccantiBrett WilliamsMauro LombardoBrian BieberMax Lennart EcksteinBrian CarsonMaxime PellegrinBrian D. RoyMaximilian Andreas StorzBrian JurewitschMaximilian Joseph BrolBridget K. BiggsMaximilian ZeydaBrigitte DahmenMay Portuguez CastroBrijesh Kumar SinghMaya KamaoBrittany HowellMayank ChoubeyBritteny HowellMayur ParmarBronisława Skrzep-PoloczekMd Abdul WazedBruna GuidaMd AdnanBruno EtoMd Ashik UllahBruno Oliveira Da Silva DuranMd Hakimul HaqueBruno TrimarcoMd Jamal UddinBryan M. GannonMd Nazmul Hasan ZilaniBryan MathisMd Nur HossainBryant AllenMd. Abdul HannanBuckley McCallMd. Al MamunBulbul AhmedMeden F. Isaac-LamBülent KilitMeehee ChoBumhee YangMeena ChellaiahBumjo OhMeerambika MishraBuyun TangMegan JarmanByaruhanga CharlesMegan M. ZeringueByeongwoon SongMegan ParkerByung-Soo KooMehdi BenkahlaC. Korcan AyataMehdi PasalarC. M. BotelhoMehdi SakkaCailu LinMehmet Ali KobatCai-Ning ZhaoMehmet KocakCaio ReisMehmet SatarCaireen RobertsMehraj AhmadCaisheng WuMehrez ElnaggarCaitlin M. LoweryMehrnaz ShoushtarianCaitlyn PlacekMehtap SahinerCălin Gabriel FloareMei Hui LiuCamelia F. OroianMei PengCamelia UngureanuMei Shan LiewCamila Renata CorrêaMei ZhaoCamilla CattaneoMeichen PanCamilla FiorindiMei-Chin LuCamilla PasternackMeiHua YuanCamille BrehinMeike RombachCamille DesgrouasMeilian LiuCandice Allister PriceMeira MachadoCara J. WestmarkMelanie B. GillinghamCari BogulskiMelanie DupuisCarina Gabriela UasufMelanie Jean CoathupCarina I. MackMelanie KorbeliusCarine ChassainMélanie PlourdeCarla Busquets-CortésMelanie S. MatosCarla FerreriMelanie SpencerCarla GonçalvesMelanie TurkCarla Maria AvesaniMelinda ManoreCarla MartinsMelisa Medina-RiveraCarla MasalaMelissa Ann TheurichCarla MoranMelissa Anne SuterCarla NoninoMelissa García-CaballeroCarla PinheiroMelissa MarianaCarla PollastroMelissa PuppaCarla QueirosMelissa ThoeneCarla TaddeiMeng ZhuCarles CantóMeng-Che HsiehCarlo CinqueMeng-Che TsaiCarlo La VecchiaMengmeng JiaCarlo TicconiMeng-Tsan ChiangCarlos Bousoño GarcíaMenicanti LorenzoCarlos Dias PereiraMenno T. C. PruijmCarlos Jiménez-CorteganaMercè GinerCarlos LifschitzMercedes LovrecicCarlos Méndez-MartínezMeriem OuniCarlos Pérez-MonterMesfin TadesseCarlos Portugal-NunesMeta MahendradattaCarlos RosadoMette Frahm OlsenCarlos Sandoval-CastroMi ZhouCarlos VasconcelosMiae DooCarlota Gómez-RincónMicah B. LeshemCarlotta GirominiMicah BattsonCarmela Maria MontoneMichael A. LeaCarmelo La RosaMichael Abou-DaknCarmen BalanMichael AuerbachCarmen F. Navarro-PérezMichael B. PapahCarmen Gonzalez-BarreiroMichael Bording-JorgensenCarmen JochemMichael ButlerCarmen López SánchezMichael FreemarkCarmen María Sarabia-CoboMichael HeneinCarmen Mayolas-PiMichael J. BarrattCarmen Ortega SantosMichael J. BrennerCarmen Patino-AlonsoMichael J. RyanCarmen Pérez-RodrigoMichael L. KuehtCarmen SanzMichael LeanCarmine FinelliMichael LentzeCarmine GazzarusoMichael LichtenauerCarmine IzzoMichael McKinleyCarol WhamMichael MillerCarola SeveriMichael R. KozlowskiCarole BrosseauMichael R. Mac ArthurCarolien Annika Van Loo-BouwmanMichael RigbyCarolin KolmederMichael RobertsCarolina De CiuceisMichael SackCarolina MuscoliMichael SchirmerCarolina Sánchez-RodríguezMichael StalveyCaroline EvansMichael W. GreeneCaroline J. SmithMichael WirthCaroline KissMichaela ŠiklováCaroline S. StokesMichał BronikowskiCaroline UmMichał BrzezińskiCarra A. SimpsonMichal ChmielewskiCarsten CarlbergMichal CzaplaCarsten TheissMichał CzaplaCaryn ZinnMichał GondekCasper SchalkwijkMichal JurášekCassamo Ussemane MussagyMichal KorostynskiCassie S. MitchellMichał MajewskiCatalina Amadora PomarMichał OczkowskiCatalina Valdés-BaizabalMichal OrdakCatarina LindqvistMichał RabijewskiCatarina MatiasMichal S. KarbownikCaterina AnaniaMichał ŚwiecaCaterina BonfiglioMichał ZarobkiewiczCaterina LombardoMichał ZimeckiCaterina Maria GambinoMichal ZmijewskiCaterina MianMichel BurnierCaterina PalleriaMichela BuricoCatharine RossMichela FerrucciCatherine ChanMichela PerroneCatherine ChristieMichele CiullaCatherine McDonaldMichele D’AttilioCatherine MichelMichele Di CosolaCatherine NortonMichele GolinoCatherine ThorntonMichele MalaguarneraCatherine WallMichele NavarraCatherine Weikart YeckelMichele UmbrelloCatherine YzydorczykMichele ZiniCathriona MonnardMichel-Edwar MickaelCayetano Medina-MolinaMichelle AverillCecile Bienboire FrosiniMichelle HarvieCecilia Alegado JimenoMichelle Ji Yeon YooCecilia ManciniMichelle JudgeCecilia VarjuMichio HongoCecilia Zavala-TecuapetlaMichiru HirasawaCecilie DahlMicol AvenaliCélia C.G. SilvaMieszko WięckiewiczCelia GarauMigle BacevicieneCéline TiffonMiguel A. Montero-AlonsoCeres M. Della LuciaMiguel Ángel Rojo TiradoCésar Chaves OiveiraMiguel Angel RubioCesare CerriMiguel León-SanzCesare SirtoriMiguel MaestroCesarettin AlasalvarMiguel MontoroChad HancockMiguel RaimundoChadin KulsingMiguel RibeiroChaitanya DendeMiguel Ruiz-CanelaChalermpong SaenjumMiha LucovnikChalida NiamnuyMihaela CotârlețChampa WijekoonMihaela SaracilaChandra Sekar Chandra MohanMihaela VladChandrakala Aluganti NarasimhuluMihai CenariuChandrasekhar KuppamMihai CovasaChang Joo LeeMihail Lucian BirsaChang XuMihail MitovChang Yu HongMihalj PošaChanggui LiMijat BožovićChangling HuMika MatsuzakiChang-Myung OhMike ReidChangqi LiuMikhail M. Vorob’evChangqing SunMikhail Viktorovich KorokinChangsoo KimMikio HayashiChang-Wei HsiehMilan HolečekChantal StheneurMilena ŚciskalskaChan-Yen KuoMilena TomanicChao DongMili DasChao MaMilica MirkovićChao NiuMilica PavlicevicChaoyi DengMilka DonchinCharalampos ProestosMilka MilevaCharalampos SiristatidisMiloš HitkaCharareh PourzandMiloslav HronekChariklia Tziraki-SegalMin Heui YooCharis LiapiMin XieCharlene B. Van BuitenMina DesaiCharlene ShoneyeMina JoCharles B. LovelyMina LeeCharles OkpalaMinakshi GandhiCharles VerneyMinami SugimotoCharlotte De GeyterMindy Millard-StaffordCharlotte Erlanson-AlbertssonMing Hsien TsaiCharlotte JaiteMing YangCharlotte LawsonMingbang WangCharlotte M. WrightMing-Fu WangCharoula NikolaouMing-Ju ChenCharuta AgasheMing-Jun TsaiChatchaporn UraipongMing-Kuem LinChen HuMing-Yu LungChen Huei LeoMingyu YeChen YangMinhao XieCheng-Maw HoMin-Hui LiCheng-Shun LuMin-Hyun KimChengwen SunMin-Sun KimChengxue ZhongMiodrag JanicCheng-Yang HuangMir Faeq Ali QuadriChenqi TaoMira HorváthováCherubino Di LorenzoMiranda DallyCheryl CeroMircea-Catalin FortofoiuCheryl Der AnanianMirco VaccaCheuk Lun LiuMirela Baus LončarChia-Fen TsaiMirela FrandesChia-Feng KuoMirela TiglisChia-Kuei LeeMirey KaravetianChianju JongMiriam Casares-LópezChiao-Ming ChenMiriam PikkemaatChiao-Nan ChenMirjam MočnikChiara AnnunziataMirjam SchuchardtChiara BegliominiMirjana StojkovićChiara BernardiniMirjana TurkaljChiara CerlettiMirko Di RosaChiara GioiaMirko MarinoChiara Maria GranaMiroslav BarančíkChiara MilaniMiroslav FišeraChiara MonachesiMiroslav JůzlChiara NovielliMiroslava AtanassovaChiara Paola ZoiaMirta CrovettoChiara RossoMirta MilicChiara VolaniMirto FolettoChia-Te LiaoMirza R. BaigChia-Wei ChengMisagh FaezipourChi-Chang HuangMisbahuddin M RafeeqChieh-Yu LinMisha D. P. LuyerChien-Hsieh ChiangMitali TambeChien-Hsing LeeMitchell E. ZaplatoschChien-Hsun HuangMitchell F. RoitmanChien-Hung LeeMitsue NishiyamaChien-Li ChenMitsue SanoChien-Ming ChaoMitsuru FukudaChien-Ming HsiehMitsuru NakazawaChien-Te LeeMitsuru OhishiChien-Yu LinMitsuya YamakitaChih-Chien SungMitsuyoshi UrashimaChih-Hsiang ChangMitsuyoshi YoshidaChih-Hsin TangMiyoung SuhChih-Hung GuoMladen AndjićChih-Jung YehMoacir MarocoloChih-Li LinMohamad KhalilChih-Ming LinMohamad MotevalliChihua LiMohamed AbdelwahabChih-Yao HouMohamed Al-FatimiChih-Yen ChenMohamed AliChikako HaraMohamed ElmonemChing-Cheng ShenMohamed SallaChing-Chung HsiaoMohammad AkmalChing-Sung LeeMohammad AkramiChing-Yi ChengMohammad BagherniyaChing-Yi TsaiMohammad Hassan HodrojChi-Nien ChenMohammad MehdizadeChin-Kun WangMohammad QneibiChin-Lon LinMohammad Radwanur R TalukderChin-Man WangMohammad Rostami-NejadChinten J. LimMohammad RostampourChirag JainMohammad ZamaniChis Adriana AureliaMohannad QazzazChiung-Huei PengMohd AdilChloe PanizzaMohd Helmy MokhtarChoon Guan LimMohd ImranChris KourekMohd Nazam AnsariChris PaciMohd RaufChrissoula VoidarouMohd Razif ShahrilChristelle Bou-MitriMohit MehtaChristian DualéMohsen KerkeniChristian FleischhakerMohsen MazidiChristian Griñán-FerréMoïse CoeffierChristian JungnickelMoises DiagoChristian RitzMoisés Tolentino B. Da SilvaChristian SelingerMojca KorošecChristian SinaMojtaba KavianiChristian TennertMojtaba ShafieeChristian Von LoeffelholzMojtaba VaismoradiChristian WiegMona BoazChristian WrightMona Hamdi HaronChristina MavrogianniMonica BaroneChristina McKercharMonica BoirivantChristine BoschMónica CostaChristine L. ParrMonica GalleanoChristine Lux‐BattistelliMonica GuglielmettiChristine TaylorMonica Kazlausky EsquivelChristoforos GiannakiMonica Livia GheorghiuChristoph CastellaniMonica Maribel Mata-MirandaChristoph JochumMonica MironescuChristoph KurzMonica NavarroChristopher BellMonica TarceaChristopher Chukwudi OkonkwoMonica TrifChristopher FahsMonica ValentovicChristopher L. CoeMonica ZeseChristopher P. MattisonMonika A. ZielińskaChristopher PapandreouMonika BarbaricChristopher R. GustafsonMonika BronkowskaChristopher RogersMonika GargChristopher StremmelMonika GrabiaChristopher VannMonika JachChristos KontogiorgisMonika Karczewska-KupczewskaChristos PapaneophytouMonika KassayováChrysoula PitsouliMonika KosmalaChuan LiMonika LesickaChuang-Yuan ChiuMonika Les̈kiewiczChuanhai ZhangMonika Michałowska-SawczynChung Yu ChenMonika MielusChung-Han HoMonika SzulińskaChung-Ho E. LauMonique AucoinChungho LauMonique FrancoisChung-Ming ChenMonique LeMieuxChung-Tung J. LinMonsurul HoqChun-Jung ChenMontserrat Esteve RàfolsChun-Kuang ShihMorgan FullertonChunlei LiMorium BablyChunling HuangMorteza HaghiriChunmei WangMoshe FrenkelChunsheng WuMoshe RubinChun-Yao YangMossab Y SaeedChun-Yu ChenMostafa BorahayChun-Yu LinMozaniel OliveiraChun-Yung HuangMrigya BabutaChu-Yu HuangMuhamed FočakCicero ArrigoMuhammad Ajmal ShahCidália Almeida PereiraMuhammad Bilal SadiqCidália D. PereiraMuhammad IrfanCíntia PêgoMuhammad N ZahidCinzia FerrarisMuhammad NadeemCinzia GiacomettiMuhammad NawazCiosek ŻanetaMuhammad UmairCiriana OrabonaMuhammad Waheed IqbalCiro EspositoMuhammad ZubairClaire BerticatMuhammed Sedat SakatClaire JohnsonMukesh KumarClair-Yves BoquienMukesh Kumar SriwastvaClara BalsanoMukul VijClara GrossoMumtaz AnwarClara Velázquez-SánchezMunekazu KomadaClarence E. GrimMussie MsghinaCláudia AfonsoMustafa Sait GonenClaudia AltamuraMustafa ShukryClaudia Di GiacomoMustapha Umar ImamClaudia Ferreira Da Rosa SobreiraMuthukumar KannanClaudia GenoveseMuthukumaran JayachandranClaudia HunotMutsuaki EdamaCláudia Jacques LagranhaMuzzamal HussainClaudia LoretiMyeong-Hyeon WangClaudia MancaMyoung-Sook LeeClaudia Maria BalzarettiMyra ConwayClaudia Muhle-GollMyriam AbboudClaudia SenglerMyriam Van WinckelClaudia Talero-GutiérrezMythily SubramaniamClaudia TregnagoN. Jeelan BashaClaudia VetraniNabeel AlmotairyClaudine IrlesNadia BadolatiClaudio AccianiNadia CalabrisoClaudio BucoloNadia NajafiClaudio De SimoneNadia RiveroClaudio ImperatoriNadinne RomanClaudio RomanoNaeimeh Atabaki-PasdarClaudiu MorgovanNafisa JadavjiClaus ChristophersenNagako OkudaClemens RöhrlNagham Mahmood AljamaliClemens Von SchackyNagwan SalehClifton AddisonNahal HabibiClifton M. CareyNahed HussienClivel CharltonNa-Hyung KimColin McCoinNai-Kuei HuangColleen Clarkin SchreyerNaim KhanColumba De La ParraNaima G. Cortes-PerezConcepció AmatNajat YahiaConcettina CappadoneNakata YoshioCong XieNak-Kyun SoungConnie M. WeaverNamhee KimConstantin CerbuNan ShangConstantina NasopoulouNana LiuConsuelo Pedrón-GinerNana ShinozakiCora M. BestNanae TanemuraCoral HanevoldNancy King ReameCoral SanfeliuNanda MandavaCorina Aurelia ZugravuNanette Stroebele-BenschopCorina PienarNan-He YoonCorinna EngelNaohisa ShobakoCorinna GeislerNaoki NakagawaCorliss Ann O’BryanNaoki ToyamaCornelia AmalineiNaoki YamamotoCornelia BalaNaomi KakoschkeCornelia MirceaNaoto HashimotoCornelia WiechersNaoya KishikawaCornelie Nienaber-RousseauNaoyuki TaniguchiCorrado RomanoNapoleon WaszkiewiczCortés-Castell ErnestoNaro OhashiCory J. GreeverNaroa KajarabilleCosima D. CalvanoNaser AlsharairiCosmin CituNasir KhanCostanza VaresioNassib B. BuenoCraig A. FriesenNatalia A. MuralevaCraig L. FrankNatalia AlzaCreswell EastmanNatalia BourguignonCristal HillNatalia Di PietroCristián A. AmadorNatalia DrabińskaCristian ÁlvarezNatalia Duque-WilckensCristian Del BoNatalia GeppeCristian FurauNatalia I. KuryshevaCristian Iulius SurcelNatalia K. BelishevaCristian SandovalNatalia KasicaCristiana CalicetiNatalia KordulewskaCristiana De MusisNatalia KurhalukCristiana PiccoNatália MartinsCristiane AguiarNatalia MaximovaCristiane KochiNatalia Nowacka-JechalkeCristiano CapursoNatalia PawlasCristiano J. De AndradeNatalia Pérez-SánchezCristina BarosaNatalia SoshnikovaCristina Bianca PocolNatalia TrpchevskaCristina Castejón-RiberNatalia Viktorovna NizyaevaCristina GiannattasioNatalija Ursulin-TrstenjakCristina González-DíazNataliya KolosovaCristina Hanganu-BreschNataliya YaglovaCristina Manuela DrăgoiNatasa Fidler MisCristina Maria SenaNataša Marčun VardaCristina Martin-SabrosoNatasa MilicCristina Mihaela LacatusuNatascia BrondinoCristina MolinerNatasha LelijveldCristina MonteiroNathalia PadillaCristina PolitiNathalia PizatoCristina Rivera-PicónNathalie VionnetCristina Rosell-ValleNathaniel VacantiCristina RussoNathaniel WeygantCristina SoaresNavarro Gómez NoeliaCristina SobacchiNaveen SharmaCristina TruzziNavid AbedpoorCristina VassalleNavid Jubaer AyonCristina-Mihaela LăcătușuNavid RabieeCristobalina MayorgaNeda O. ĐorđevićCrystal Haskell-RamsayNedaeinia RezaCui-Ping FengNeeraj Kumar SethiyaCunchuan WangNeil ClarkeCurtis D. KlaassenNeil YoungsonCynthia BarreraNejc UmekCynthia BlantonNela Nedić TibanCyril D. MamotteNelson GomesCyril NicolasNelson Gutiérrez GuzmánCyrus MotamedNemanja LakicevicDae-Sung LeeNemanja VujicDagmar DzúrováNemat AliDagmara ZłotkowskaNemesio Villa-RuanoDaiji NagayamaNeumann Felix AlexanderDaisuke AriyasuNeuza Mariko Aymoto Aymoto HassimottoDaisuke KobayashiNevena IvanovicDaitoku SakamuroNeža MajdičDale E. SchoellerNicholas D. LudenDâmaris SilveiraNicholas J. OllberdingDamien LannoyNicholas Mascie-TaylorDamir ZubacNicholas SiscoDan DumitrascuNicholas V. C. RalstonDan JiangNick KouttabDan JonesNicklas BrustadDan Linetzky WaitzbergNicola CirilloDan VoiculescuNicola LaforgiaDana BadauNicola LoweDana Teodora Anton-PaduraruNicola MontemurroDanh V. NguyenNicola OreficeDaniel A. MedinaNicola SponsielloDaniel AmundNicolae BacalbașaDaniel Antonio De LuisNicolae GicaDaniel BecqueNicolai Jacob Wewer AlbrechtsenDaniel BonthiusNicolas BergerDaniel D. SavageNicolas ClarkDaniel Das Virgens ChagasNicolas GuyotDaniel DziobNicole EricksonDaniel EyraudNicole GilbertsonDaniel Gabriel PonsNicole PizzorniDaniel Henrique BandoniNicole Sommer GrünDaniel J. HoffmanNicoleta AntonDaniel JanitschkeNicoletta Di SimoneDaniel MauvoisinNicoletta PellegriniDaniel MeresteinNicolette W. De JongDaniel N. WarshawskyNicos MaglaverasDaniel P. PotaczekNida I. ShaikhDaniel P. ZalewskiNidhish FrancisDaniel Robert SchlattererNiek F. CasteleijnDaniel ŚlęzakNieves Luisa Gonzalez GonzalezDaniel TimofteNieves Palacios Gil-AntuñanoDániel TöröcsikNighat NoureenDaniel WestNihar KinarivalaDaniela BuchaimNika PavlovićDaniela CaccamoNikhil Suresh BhandarkarDaniela Caetano GonçalvesNikol SulloDaniela CarlisiNikola Hadzi-PetrushevDaniela CovinoNikola StojanovicDaniela De BartoloNikolaj TravicaDaniela ErbaNikolaos A. ChrysanthakopoulosDaniela FreitasNikolaos AntonakopoulosDaniela GabbiaNikolaos PitsikasDaniela HaluzaNikolaos SiderisDaniela HampelNikolaos SolomakosDaniela HanganuNikolay LobusDaniela MartiniNikoleta PrintzaDaniela MarzioniNikolina Jukić PeladićDaniela MenichiniNikos GkraniasDaniela MorniroliNikos ViazisDaniela PintoNilanjana BanerjeeDaniela PodleckaNilesh J. PatelDaniela RodriguesNils BömerDaniela RovitoNimbe TorresDaniela UbertiNina Kacjan MaršičDaniela Viramontes HornerNina Van DykeDaniele MonzaniNingning WangDaniele NovielloNipith CharoenngamDaniele NucciNipun SainiDanielle GreenbergNirajan ShresthaDanielle R. StevensNirupama ShivakumarDanielle RobertsonNisha SinghDanijela Ristić-MedićNishel Mohan ShahDanijela TasicNitin KapoorDanilo Escobar-AvelloNiva ShapiraDanilo MilardiNizar TliliDanit ShaharNoa Ofek-ShlomaiDanko MiloševićNoala MihanDantong WangNobuaki MichihataDanuta GajewskaNobuo NishiDanuta Rosolowska-HuszczNobuyuki KimuraDany Munoz-PintoNobuyuki SakaiDaphna DrorNobuyuki SakayoriDarel Wee Kiat TohNoé OntiverosDarie CristeaNoelia González-GálvezDarinka AckovaNoelle Elizabeth YoungeDario CalafioreNoemí Echegaray SuárezDariusz NowakNoemí TomsenDariusz WłodarekNoereem MenaDarrell CockburnNoha NasefDarshan KelleyNoor BuchholzDavid A. AugustNorbert StefanDavid A. TulisNorbert TripoltDavid BeversdorfNoreen F. RossiDavid BrooksNoreen RossiDavid CoelhoNorihisa KatoDavid ConversiNoriko I. TanakaDavid DaguetNoriko TakegaharaDavid DaniepourNorio SogawaDavid DoraNoriyasu OtaDavid H. AlpersNoriyuki KajiDavid HeberNoriyuki NakayamaDavid Hung-Tsang YenNoriyuki YanakaDavid J. MartinoNorizzati AmsahDavid José Casimiro AndradeNorrie PearceDavid KeaneNoushin MohammadifardDavid KumpNunes Farinha Carlos ManuelDavid MaNuno MoraisDavid MandelbaumNunzio CardulloDavid MelaNupur DasDavid MooreNurdan GüldikenDavid Morales-AlamoNuria CasalsDavid N. CoxNúria FarréDavid P. GallowayNuria SalazarDavid Ramiro-CortijoNur-Vaizura MohamadDavid RamonetOak-Hee ParkDavid S. GoldfarbOana A. ZeleznikDavid SeversOana Emilia ConstantinDavid SmithObaid AfzalDavid StuckiOck K. ChunDavid Varillas DelgadoOctavian AndronicDavid W. DodingtonOctavian EnciuDavid WhiteOdile ViltartDavide BorroniO’Donnell EmmaDavide BrottoOfélia AnjosDavide Giuseppe RibaldoneOfer GoverDavide LeardiniOhsawa SuguruDavide SistiOk-Kyung KimDavina Camargo Madeira SimoesOkyaz EminagaDavit PipoyanOlah HakimDawid GawlińskiOlaia Lucas-JiménezDawid MadejOlasunkanmi A. John AdegokeDawit Albieiro Pinheiro GonçalvesOleg KaradutaDayong ShiOlena PrykhodkoDe Keukeleire Denis D.Olga Alekseevna GromovaDebabrata DasOlga BarbarskaDebananda GogoiOlga Catalina RodriguézDebasis BagchiOlga GoloubevaDebasish Kumar DeyOlga PagonopoulouDebo DongOlga TsaveDebora BizzaroOlga TsivilevaDébora Cerdá-BernadOlga V. BelyaevaDebora Farias Batista LeiteOlga Witkowska-PilaszewiczDébora Luana Ribeiro PessoaOlga ZaborinaDebora MacisOliver MeixnerDébora Martins Dos SantosOliver Mendoza-CanoDeborah A. FrankOlivera GalovićDeborah KerrOlivia LapentaDecha SermwittayawongOlivier BraissantDeepak KumarOlivier BruyèreDeepak Kumar KhajuriaOlivier GasserDeepika GargOlivier LaprevoteDeiana RomanOliwia Gawlik-KotelnickaDelia TitOliwia Zakerska-BanaszakDelicia Shu Qin OoiOlja ŠovljanskiDelphine MaurelOllie Yiru YuDemin CaiOmar AlhajDénes MolnárOmar DaryDenes SteflerOmar Guzman-QuevedoDenis A. BabkovOmar JamilDenis FouqueOmar KujanDenis PicotOmbretta RepettoDennis AdjepongOmid MirmosayyebDennis CladisOmni CassidyDennis E. JewellOmobolanle (Bola) OloyedeDennis SavaianoOndřej ŠedaDer-Yuan ChenOndrej SlanarDesiree Lopez-GonzalezO’Neill LyndaDesirée Mena-TudelaOran KwonDesiree TandeOriol Abellan-AynesDesiree WandersOriol RangelDeven PatelOrjena ŽajaDevojit Kumar SarmaOrtega Moreno LorenaDewi GuellecOsamu KushidaDhananjay YadavOscar CastilloDiana AntalOsmar Antonio Jaramillo-MoralesDiana Drago-GarcíaOsmindo R. Pires JúniorDiana LelliOtilia BobisDiana MartinsOtilia MargineanDiana SilvaOto HanušDianbao ZhangOttavia Eleonora FerraroDiane BunnOussama SaidiDiane DepkenOuti PellonperäDiane M. DellaValleOvidiu TitaDiane StadlerOwczarek DanutaDibya PandaÖzgül EkmekçiDidier QuilliotOzgur EkenDiego A. RojasP. Bryant ChaseDiego Alzate CorreaPablo Blanco Martínez De MorentinDiego AraunaPablo Jesús Marín-GarcíaDiego Casas-DezaPablo Ureña-TorresDiego F. TiradoPadma MaruvadaDiego La MendolaPäivi E. KorhonenDiego Marcos-PérezPalanikumar BalasundaramDiego MarquésPalanisamy NallasamyDiego Marqués-JiménezPalaniyandi KaruppaiyaDiego MartinezPalavalasa SravyaDiego PeroniPalma ScolieriDiego RaimondoPamela DysonDieter LütjohannPamela PignateliDietmar EnkoPamela SenesiDietmar KrautwurstPanagiota G. PietriDihogo De MatosPanagiota MitrouDimitra Rafailia BakaloudiPanagiota XaplanteriDimitrios A. AnagnostopoulosPanagiotis AthanassiouDimitris A. GougoulisPanagiotis I. GeorgianosDimitris SkalkosPanagiotis KanellosDimitris TsikasPanagiotis PeitsidisDimitris TsoukalasPanagiotis TheofilisDimitris ZacharoulisPanagiotis TsiartasDina El-KershPanagiotis TsikourasDina Rešetar MaslovPanda SantoshDina SimesPaniz JasbiDinesh BabuPanteleimon GiannakopoulosDingbo LinPantelis ZempekakisDing-Han WangPaola BagnoliDinithi PeirisPaola CicatielloDiogo CunhaPaola CorsettoDiogo FonsecaPaola Di PietroDiogo Thimoteo Da CunhaPaola FiniDiosey Ramon Lugo-MorinPaola Gauffin-CanoDipankar AshPaola GiordanoDirce MarchioniPaola Maura TricaricoDitte HansenPaola NardoneDivya SinhaPaola NavarreteDmitriy SmolenskyPaola PalestiniDmitry KarpovPaola QuaresimaDolores CorellaPaola RoggeroDolores D. GuestPaola VendittiDolores MarrodanPaolo CiampaliniDomagoj VidosavljevicPaolo ContieroDomenic MarbaniangPaolo DorettoDomenica MangieriPaolo Emilio PudduDomenico AzzolinoPaolo GabrielliDomenico CucinottaPaolo GentileschiDomenico PalumboPaolo MagniDomenico SantoroPaolo MarzulloDomenico TricaricoPaolo Matteo AngelettiDomingo RamosPaolo MeneguzzoDominic AugustinePaolo MonardoDominik DłuskiPaolo NataleDominik ZimonPaolo PolidoriDominika GlabskaPaolo SeverinoDominika GłąbskaPaolo TrucilloDominika GuzekPaolo Usai SattaDominika SalamonPao-Yun ChengDominika SkolmowskaPapasani Venkata SubbaiahDomitilla MandatoriParamjit TappiaDon A. DaviesParaskeui DetopoulouDon GreenParaskevi MitliagkaDonald C. ColePascal CrennDonald IngramPascal P. CrennDonald MessnerPascal PeraldiDonatella Degl’innocentiPasqua Anna QuitadamoDonatella PontiPasquale AuricchioDonato AngelinoPasquale CaldarolaDonato CalabriaPasquale DolceDong Keon YonPasquale Fabio ProvenzanoDong LiuPasquale MoneDong Seon KimPasquale ParibelloDong Soon ImPatrice MarquesDong Wook LimPatricia A. MiguezDong Wook ShinPatrícia BotelhoDong-Hao WangPatricia De Carvalho PadilhaDong-Hee RyuPatricia Gálvez EspinozaDong-Keun SongPatricia HuebbeDongliang WangPatricia L. MitchellDongshan ZhuPatrícia PadrãoDongwei ZhangPatricia Pérez-MatuteDonna M. WinhamPatricia RegalDonovan ArguetaPatricia SheeanDorin GheorghePatrick BradshawDorin IonescuPatrick CoppensDorinda Marques Da SilvaPatrick DansetteDoris KretzschmarPatrick G. ArndtDoris RusicPatrick H. DesseinDorit AvniPatrick H. J. HemmerDorota Haznar-GarbaczPatrick HerrDorota KoziełPatrick PoucheretDorota SulejczakPatrizia GenaDorota Szostak-WęgierekPatrizia PasanisiDorota Walkowiak-TomczakPatrizia RestaniDorota WianowskaPatrizia RisoDorota WultańskaPatrizia TodiscoDorothea HaasPatrizia ZaramellaDouglas BurrinPatrizia ZavattariDouglas MatthewsPatrizio BolleroDragan MilicevicPatrycja KupnickaDragana KomnenovPatryk LipinskiDragana KopanjaPatryk TarkaDrago BesloPatxi León-GuereñoDraško PavlovićPaul BatchelorDrew BudnerPaul C. RogersDu YuchunPaul ClaytonDulce Maria MinayaPaul DawsonDulce MinayaPaul Eze EmeDulcenombre Gómez-GarrePaul GardDumitru CiolacPaul H. M. HarrisonĐurđica AčkarPaul J. CurrieDuska Martinovic KaliternaPaul S. AmieuxDuygu AğagündüzPaul S. HafenDyana OdehPaul SchoenhagenE. Maruthi PrasadPaula Alexandra A.B. LopesEbba NexoPaula Clara SantosEddy J. DavelaarPaula DworatzekEdel KeaveneyPaula M. MillinEder Carlos Rocha QuintãoPaula MosińskaEdgar Torres-MaravillaPaula NormandoEdgard DelvinPaula NuñezEdgardo EtchezaharPaula PereiraEdit HermeszPaula PintoEdith AranyPaula SilvaEdmundo M. Mercado-SilvaPaula Skye TallmanEdmundo Rosales-MayorPaula VerissimoEdna NavaPaul-Alexandru PopescuEdna Regina AmantePaulina Correa-BurrowsEdoardo BraunerPaulina DumnickaEdoardo Maria MuttilloPaulina MertowskaEdoardo MuratorePaulina NowaczykEdsaul Emilio Perez-GuerreroPauline DouglasEdson AntunesPauline RaoulEdson SilvaPauline RebouillatEduard BaladiaPaulo MatafomeEduard IsenmannPavel KopelEduardo Augusto Fernandes NilsonPavol FarkašEduardo J. SimoesPawan Kumar RautEduardo Pandolfi PassosPawan Sirwan FarisEduardo Vieira NetoPawel BieganowskiEduardo Yoshio NakanoPaweł BogdańskiEdurne SimonPawel BrylaEdward F. CoylePaweł BryłaEdward FitzgeraldPaweł GaćEdyta BrzóskaPaweł GlibowskiEdyta DziadkowiakPawel KielaEdyta Gendaszewska-DarmachPaweł PiepioraEfrosyni G. NomikouPaweł RamosEfstratios-Stylianos PyrgelisPaweł SiudemEfthymia PapadopoulouPaweł WięchEgeria ScodittiPayam BehzadiEggersdorfer ManfredPedram ShokouhEgija ZauraPedro AraujoEglantine BallandPedro BullonEiji MunetsunaPedro Ferreira-SantosEirini BathrellouPedro Gil-MadronaEirini ChristodoulouPedro Gonzalez-MenendezEirini MamalakiPedro José Tauler RieraEirini PanagopoulouPedro ManonellesEisuke KatoPedro Manuel Rodríguez-MuñozEitaro KodaniPedro MoreiraEkaterina V. ZubarevaPedro PellicerElaine McCarthyPedro SaavedraEleanore V O’NeilPedro SouzaEleftherios K. AlissandrakisPeggy PolicastroElena Alonso-ApertePei-Fen YenElena BernadPei-Fung WuElena Crehuá-GaudizaPeihua JiangElena DomínguezPeihua MaElena DonettiPei-Ling HsiehElena GangitanoPei-Luen LuElena HadjimbeiPei-Yi ChuElena KistanovaPei-Ying PaiElena M. Yubero-SerranoPekka MäättänenElena Martínez-SanzPellegrino RossiElena MontiPenelope IoannidouElena Rodríguez RodríguezPeng JiElena SchmidtPeng WangElena SuccurroPengbiao XuElena TarcaPengfei XuElena UribePer Medbøe ThorsbyElena V. GirichPer SöderstenElena V. IndukaevaPernille HolmagerElena V. ProskurninaPernille Tveden-NyborgElena-Daniela GrigorescuPeter AbujaElena-Suzana Biriș-DorhoiPéter ÁcsEleni AndreouPeter C. FritzEleni RebelosPeter Christopher KonturekElenilson De Godoy Alves FilhoPeter David WagnerEleonora LaiPeter F. FennessyEleonora TruzziPeter FineElettra MancusoPéter FodorEleuterio A. Sánchez-RomeroPeter FrostElia RanzatoPeter J. AggettEliano CascardiPeter J. CurtisElif Inan-ErogluPeter John JervisElif YildizPeter KerrElin ChorellPeter LemonEline Van Der BeekPeter MachnikElio F. De PaloPeter MassányiElisa BorsaniPeter PikoElisa CalabriaPeter R. KvietysElisa CeccheriniPeter TiidusElisa GiuntiPeter WitzgallElisa Holmlund-SuilaPetjon BallcoElisa MazzaPetr ZacekElisa ZavattaroPetra Bauer-KreiselElisabet A. ForsumPetros DinasElisabet Navarro-TapiaPetru JitaruElisabeta BotezPeyman RezaieElisabeth JacobsenPhil AyersElisabeth RiegelPhil ChilibeckElisabeth S. GruberPhil MayElisabeth SchnoyPhilip Brandon BusbeeElisabeth Sornay-RenduPhilip CalderElisabetta AntonelliPhilip HouckElisabetta CaiazzoPhilip JakemanElisabetta MurruPhilip PrinzElisardo VasquezPhilipp SchererEliška SovováPhilipp SchuetzElitsa AnanievaPhilippe ChauveauElitza P. Markova-CarPhilippe HujoelElizabeth A. ParkerPhilippe LesnikElizabeth A. WhitcombPhilippe MarteauElizabeth Aparecida Ferraz Da Silva TorresPia Karlsland AkesonElizabeth C. CottrellPia Lopez-JornetElizabeth C. PenickPia VidalElizabeth D. NobmannPiergiorgio BolascoElizabeth FragopoulouPiero AmodioElizabeth GollubPiero ChirlettiElizabeth J. JohnsonPieter De LangeElizabeth RacinePieter Jj SauerElizabeth S. FernandesPietro Amedeo ModestiElizheeba Christie AbrahamPietro FormisanoElke NaumannPietro MorroneElke OetjenPilar Carranza-RosalesElke TrautweinPilar Pérez-RosEllen Amanda StruijkPilar SanchisEllen HertzmarkPing K. YipEllen Siobhan MitchellPing SongElom Kouassivi AglagoPing YaoElpis MantadakisPing ZhangElpis VlachopapadopoulouPing ZhouElsa UribePing ZouElsa VascoPing-Fing ChiuElsa VitalePing-Hsun WuElsayed MehanaPin-Kuei FuElumalai SanniyasiPinto-Almazán RodolfoElzbieta Jarocka-CyrtaPio ContiElżbieta Jarocka-CyrtaPiotr CeranowiczElzbieta KlewickaPiotr DobrowolskiElżbieta Łodyga-ChruścińskaPiotr HeczkoElżbieta MillerPiotr JaniszewskiElżbieta PetriczkoPiotr KaczkaElżbieta Poniedziałek-CzajkowskaPiotr KonopelskiElżbieta PoniewierkaPiotr KuczeraElżbieta Sochacka-TataraPiotr KuśElżbieta Studzińska-SrokaPiotr PoznanskiEmad Al-DujailiPiotr SobierajEman E. MahmoudPishan SungEman MehannaPo-An HuEman Y. GoharPo-Jen HsiaoEmanuel K. ManesisPolina PopovaEmanuel Kennedy-FeitosaPompilia Ispas-SzaboEmanuel Loeza AlcocerPonlapat RojnuckarinEmanuela BianciardiPonnuraj Nagendra PrabhuEmanuela Gualdi-RussoPoulami DattaEmanuela Micioni Di BonaventuraPradeep PodilaEmanuela ViggianoPradeep ShuklaEmanuele RinninellaPramod GopalEmer FerroPramod KhoslaEmidio ScarpelliniPranabananda DuttaEmiel F.M. WoutersPrasad RasaneEmile LevyPrasanth PuthanveetilEmilia AlvesPrasart NuangchalermEmiliano CèPrashanth Kumar Bhusetty NageshEmilie CardonPraveen BirurEmilie ViennoisPraveen RajendranEmilien JeannotPrawej AnsariEmilio Fernández-EspejoPrawez AlamEmilio RosPredrag JovanovicEmily C. RadlowskiPreethi BalanEmily CaponePreeti ChandraEmily HoPrema VelusamyEmily J SauersPriscila Dauros SingorenkoEmily JaehnePriscilla DayEmily JohnstonPriti Prasanna MaityEmily PadhiPriviledge CheteniEmily W. FlanaganPriya Darshni ShanmugarajahEmma BorrelliPriyadarshini KachrooEmma De FabianiPriyanka SandalEmma EspinosaPriyankar DeyEmmanouela SdonaPrzemysław DomaszewskiEmmanuel BiverPrzemysŁaw ł. ZalewskiEmmanuel RineauPrzemysław TomasikEmmanuela MagriplisPugazhendhi SrinivasanEmmanuella MagriplisPuhan HeEmmanuelle RochettePuhong ZhangEmpar ChenollPuja AgarwalEmy Luiza Ishii-IwamotoPushpa Raj JoshiEnas A. Abdel-HadyPu-Ting DongEnbo MaQi QiaoEndrit ShahiniQi WangEnedina QuirogaQi ZhangEnette Larson MeyerQian LiEnkhmaa ByambaaQianfen WanEnoch A. MensahQiang FuEnquan XuQiangqiang LiEnri NakayamaQiao ShiEnric I. CanelaQin WuEnrico CollantoniQing WangEnrico GiordanQinglan DingEnrique Domínguez-ÁlvarezQing-Ping XieEnrique Méndez BolainaQing-Rong LiuEnrique Ramón ArbuésQingsen ShangEnrique Reyes-MuñozQingzhe JiaEnrique Romero-VelardeQinqin PuEnxiang ZhangQiqi ChenEnza MozzilloQiushi FengEnza SperanzaQuang Duy TrinhEnzo SpisniQuanjun LyuEraci DrehmerQuanwei ZhangEric BattagliaQuoc Cuong NguyenEric De GrootR. CermakEric E. CallowayR. Martin PayoEric GouletR. PandiselvamEric Kam-pui LeeRachael Leonn GuerreroEric Paul PlaisanceRachael TaylorEric Van BredaRachana D. ShahErica MenegattiRachana ShahErica VareseRachel CooneyErich MöstlRachel GillespieÉrick De OliveiraRachel L. WashburnErick Gutiérrez-GrijalvaRachel MurphyErick Sierra-CamposRachel RadinÉrico Chagas CaperutoRachel TanErik BehringerRachid SkoutaErik FroyenRaci KarayigitErika Aparecida SilveiraRado PisotErika CioneRadomír HyšplerErika Mariana PalmieriRadosław KempińskiErika PalmieriRadosław KowalskiErin ClarkeRadosław KujawskiErin G Reed-GeaghanRadoslaw RolaErlinda TheRadu AlbulescuErmelinda De MeoRadu BotezatuErnest E. SmithRadu C. RacovitaErsin DemirRadu ChiceaEssi HavulaRadzisław MierzyńskiEstefanía Burgos-MorónRafael Aiello BomfimEstela BlancoRafael Moreno-Gómez-ToledanoEster VitacolonnaRafael RavinaEsther CaparrósRafael Ríos-TamayoEsther Cuadrado SotoRafael Salto-GonzalezEsther ErdeiRafael Scaf De MolonEsther Ocete-HitaRafael Urrialde De AndrésEthan David CohenRafaela G. FeresinEtienne MerlinRafaella BuzzettiEttore VarricchioRafał FilipEugenia BezirtzoglouRafal KocylowskiEugenia Silva-HerzogRafel M. PrietoEugenia YiannakopoulouRaffaele CapassoEugenio ProvenzanoRaffaele PillaEugenio RagazziRaffaele SerraEun Jeong KoRaffaella CancelloEuni LeeRaffaella ComitatoEunjung KimRagai R. MitryEusebio ChiefariRaghav KaliaEva BagyinszkyRaghbendra Kumar DuttaEva ConceiçaoRaghunath SinghEva CulakovaRaghuveera GoelEva KnuplezRahmat AttaieEva M. Medina-RodríguezRahul KumarEva NüskenRaili MüllerEva PolyakRainer WirthEva S. LiuRais AnsariÉva SzabóRaj KumarEva TsaousidouRaja GanesanEvangelia AntoniouRaja Gopal Reddy MooliEvangeline MantziorisRaja Singh PaulrajEvangelos SymeonidisRajanikanth VangipurapuEvasio PasiniRajeev AuroraEvelien MertensRajesh ChaudharyEvelien SnauwaertRajesh ParsanathanEvelina Maria GosavRajiv JanardhananEvelyn Frias-ToralRajmohan DharmarajEvelyn LamyRajshri RoyEvelyn Nunes Goulart Da Evelyn N G PereiraRakeshkumar B. PatelEvert F.S. Van VelsenRalf JaegerEvert M. Van SchothorstRaluca DumacheEvertine WesselinkRaluca Maria PopEvgeny R. BojkoRam PrasadEvika KaramagioliRamachandran ChelliahEwa BalcerczakRamesh KumarEwa Bielczyk-MaczyńskaRamesh PeriasamyEwa Błaszczyk-BębenekRamez AbdallahEwa Czarniecka-SkubinaRami BoukhalilEwa DudzińskaRami S. NajjarEwa FiedorowiczRamón EstruchEwa HalickaRamona D’AmicoEwa JablonskaRan AnEwa JakubczykRandal S. StahlEwa LangeRani PallaviEwa MarcinkowskaRania MekaryEwa Marcinowska-SuchowierskaRanjana BirdEwa Miller-KasprzakRanjeet DhokaleEwa Olchowik-GrabarekRanjeev Chrysanth PulleEwa PiątkowskaRanjini SankaranarayananEwa PlantRaquel Boia﻿Ewa SicinskaRaquel Braz Assuncao BotelhoEwa SicińskaRaquel BurrowsEwa StachowskaRaquel De Cássia Dos SantosEwa TomaszewskaRaquel Lara MorenoEwa Wender-OzegowskaRaquel Revuelta IniestaEwelina GaszynskaRaquel Sanabria-De-La-TorreEwelina KrólRaquel YahyaouiEwelina Pałkowska-GoździkRareş Mircea BîrluţiuEziuche Amadike UgboguRasa JankauskieneF. Teferra TadesseRastislav JendželovskýFabian BockRataya LuechapudipornFabiana GeraciRauf AbdurFabiane Valentini Francisqueti-FerronRaul DomínguezFabien ChevalierRaúl DomínguezFabio Arturo IannottiRaúl González-DomínguezFabio CaradonnaRaul Zamora-RosFabio Di DomenicoRavi Kumar KomaravoluFabio GalvanoRavikumar K. JimmidiFabio LauriaRavikumar ManickamFábio Luiz MialheRavinder NagpalFabio MussoRavinder Reddy GaddamFabio PaceRayan Khaddaj MallatFábio Santos LiraRayaz A. MalikFabio TimeusRaymond PalmerFabrice Mac-WayRaymond Scott TurnerFabrício Eduardo Bortot CoelhoRay-Yu YangFabrizio Dal PiazRǎzvan HainǎroşieFabrizio DamianoRebeca Fernández-CarriónFabrizio GentileRebeca Monroy TorresFahad KhanRebecca BennettFahd AlhamdanRebecca CostelloFahd QadirRebecca HerzogFahimeh VarzidehReed MangelsFang ZhengRegina MenezesFangcong DongRegina WierzejskaFarah Ibrahim Al-MarzooqRegis MoreauFarhad VahidReijo LaatikainenFarid BoubredReiko NakaoFarid KhalloukiReima SuomiFarid RahimiReiner BuchhornFarideh HosseinkhaniReinhard KalbFarooq AhmedReinhard ZelenyFarooq SyedReinhold FeldmannFarren BriggsRéjean CoutureFarzad AmirabdollahianRemco OostendorpFarzan SolimaniRena KostiFatemeh Mohammadi-NasrabadiRenalison Farias-PereiraFatima MartinsRenan Dal-FabbroFátima O. MartinsRenan DanielskiFatima RivasRenata BarrosFatme AlAnoutiRenata BartesaghiFazle ElahiRenata CarneiroFederica AureliRenata FinelliFederica BranchiRenata KucieneFederica FogacciRenata Pietrzak-FiećkoFederica FrancescangeliRenata Puppin ZandonadiFederica LaguzziRenata ZandonadiFederica MaggiRené J. L. MurphyFederica PrinelliRenee KorczakFederica TonoloRen-In YouFederico BozzettiRenuka JayatissaFederico RoggioRen-Wang JiangFei MenRen-You GanFeili Lo YangReza ArezoomandanFeili YangReza JafariFelice JackaRičardas RadišauskasFelicia Ramona BirauRicardo Augusto De Melo ReisFeliciano Priego-CapoteRicardo Fernando ArraisFelipe J. AidarRicardo Rebelo-GonçalvesFelipe José AidarRiccardo FerroFelipe VadilloRichard B. KreiderFelix ChanRichard BaybuttFelix GoniRichard BolesFélix Javier Jiménez-JiménezRichard D FeinmanFemke HoevenaarsRichard HurrellFenghai GuoRichard KreiderFeng-Hua SunRichard L. BennettFenglin ZhangRichard LebaronFengna LiRichard M NilesFengqing ChaoRichard Steven PappasFerdinand HaschkeRichard ZuelligFerdinando FranzoniRickelle RichardsFerdinando PaternostroRie TsutsumiFerenc BudanRie YanagisawaFerenc VinczeRigas G. KalaitzidisFermin García-Muñoz RodrigoRika Indri AstutiFernanda RodriguesRiki TeslerFernando AzpirozRima ObeidFernando Capela E SilvaRinaldo PellicanoFernando CardonaRintu ThomasFernando GõmezRishabh C. ChoudharyFernando Gonzalez-UarquinRishabh DevFernando José Figueiredo Agostinho D’Abreu MendesRishi Man ChughFernando Luiz Pereira De OliveiraRita Cássia Coelho De Almeida AkutsuFernando MataRita Del PintoFernando Padovan-NetoRita KissFernando RamalhoRita NegrãoFernando Vargas JuniorRita PadoanFerruccio GallettiRita PolitoFilipa I. BaptistaRitesh ChimoriyaFilipa MandimRitwik DattaFilipa Mascarenhas-MeloRiwanti EstiasariFilipa VicenteRizaldy ZapataFilippo MarianoRizwan QaisarFilippo RossiRobert A. HegeleFilomena MazzeoRobert B. RuckerFiona E. HarrisonRobert BłaszczykFiona HarrisonRobert Brawura-Biskupski-SamahaFiona LimanaqiRobert D. RoghairFiona McKayRobert DiSilvestroFiroz AkhterRobert E. SmithFitri OctavianaRobert EckenstalerFlávia Aparecida GraçaRobert Emmett KellyFlavia Fondevila PeñaRobert GajdaFlavia MagriRobert HamlinFlavia MuleRobert J. BrownFlemming AndersenRobert JonesFlora N. BalievaRobert KissFlore DepeintRobert KotloskiFlorence LaiRobert M. BadeauFlorence NeymotinRobert M. SiegelFlorian K. EbnerRobert MagdaFlorin FilipRobert N. HelsleyFlorin GorunRobert P. YoungFlorina M. BojinRóbert PókaFlorin-Dumitru PetrariuRobert R. WeaverFortunato IacovelliRobert ŚmigielFotini TsofliouRobert Steven EsworthyFrances Hardin-FanningRobert Stiles ThompsonFrancesc Fusté-FornéRobert SusloFrancesc PuiggròsRobert T. MeansFrancesca ArfusoRobert W NickellsFrancesca BaldassarreRobert WardFrancesca BonominiRoberta BudriesiFrancesca CasciaroRoberta GasparroFrancesca CattoniRoberta GiordoFrancesca CianiRoberta LupoliFrancesca CrovettoRoberta MacrìFrancesca Di CaraRoberta MasellaFrancesca GarofoliRoberta OkamotoFrancesca LatinoRoberta ZupoFrancesca MeliniRoberto AquilaniFrancesca PacificiRoberto ArrigoniFrancesca PerutRoberto BelluFrancesca SarettaRoberto CannataroFrancesca ScazzinaRoberto CastiglioneFrancesca TruzziRoberto CiampagliaFrancesca ViazziRoberto CodellaFrancesco AcciaiRoberto ColluFrancesco AsciRoberto FabianiFrancesco AsnicarRoberto GramignoliFrancesco BarrecaRoberto Lo GiudiceFrancesco BimboRoberto Martínez BeamonteFrancesco BozzoRoberto Massimo CrnjarFrancesco BrascaRoberto MinieroFrancesco CacciolaRoberto S. FrancoFrancesco CadarioRoberto ScatenaFrancesco CampaRoberto ScicaliFrancesco ComacchioRobin A. WilsonFrancesco CorattiRobin DeWeeseFrancesco CoricaRobin JoshiFrancesco Francini PesentiRobin M. TuckerFrancesco Francini-PesentiRobin McClaveFrancesco GuzziRobin TuyttenFrancesco Maria CalabreseRobin WilsonFrancesco OrienteRobson De FariasFrancesco Paolo FanizziRocio BarragánFrancesco PerticoneRocío González-SolteroFrancesco PrattichizzoRocío Muñoz-HernándezFrancesco SartiniRocío Romero-CastilloFrancesco SavinoRoddy HiramFrancesco SpallottaRodney J. SnowFrancesco SpinelliRodrigo ChamorroFrancesco SurianoRodrigo Feteira-SantosFrancesco TondoloRodrigo LópezFrancesco ValituttiRodrigo Polimeni ConstantinFranchek DrobnicRodrigo Romero-NavaFrancina Lozada NurRodrigo Torres-CastroFrancine M OvercashRodrigo TroncosoFrancisca Castro MendesRodrigo ValenzuelaFrancisca EcheverríaRodrigo Vargas VitoriaFrancisco Caamaño IsornaRodrigo Wladimir ValenzuelaFrancisco FernándezRoger BouillonFrancisco GudeRoger Gutierrez-JuarezFrancisco Guillen-GrimaRoger M. KrzyżewskiFrancisco Javier López RománRogério Luis CansianFrancisco José Medina AlbaladejoRohil BhatnagarFrancisco José Rodríguez VelascoRohit JainFrancisco José Sánchez-TorralvoRokia SanogoFrancisco M. Gutierrez-MariscalRoland DickersonFrancisco Marlon Carneiro FeijóRoland GärtnerFrancisco Murillo-CabezasRoland KocijanFrancisco Reyes-SantiasRolf BosFrancisco RiosRolf TeschkeFrancisco Sandro Menezes-RodriguesRoman J. ShypailoFrancisco SolanoRoman LysiukFrancisco TarazonaRoman SmolarczykFranco CappuccioRomana VulturarFranco CervellatiRon KedemFranco MusioRon SaulnierFranco Trepat EloiRonald B. BrownFrancois LefebvreRónán DohertyFrank Blanco-PerezRonan LordanFrank E. NelsonRong ChenFrank R. GreerRong-Fu ChenFrank SchurenRongjin SunFrankena KlaasRosa AmorosoFrantisek KvasnickaRosa Anna MilellaFranziska DenglerRosa CalvelloFranziska Roth-WalterRosa Calvo-EscalonaFranz-Josef SchingaleRosa CarotenutoFred M. PaccaudRosa DivellaFreddy BrownRosa GiuglianoFrederic J TessierRosa MancinelliFreya MacMillanRosa Maria FanelliFriederike BarthelsRosa María Giráldez-PérezFriederike HolzeRosa Maria Mateos BernalFritz SieberRosa Maria Tapia-HaroFu-Chen HuangRosa PurgatorioFulvio LauretaniRosa Rodríguez-PérezFumihiko NambaRosa SuadesFuming TsaiRosalba SiracusaFung-Fuh WongRosalinda Sánchez-ArenasFurong XuRosa-Maria Guéant-RodriguezGabby B. JosephRosanna Di PaolaGabi DachsRosaria M. RuggeriGabriel EstrelaRosario MareGabriel González-ValeroRosario PastorGabriel M. PagnottiRose-Marie SatherleyGabriela AlbuquerqueRosemary StantonGabriela C. ZaharieRoser GraneroGabriela ChiritoiuRosiane Aparecida MirandaGabriela CristeaRossella DoriaGabriela Mihaela MuresanRossella GrandeGabriela NegroiuRossella TozziGabriela RiscutaRoxana Adriana StoicaGabriela Vollet MarsonRoxana LucaciuGabriele DonatiRoxana StegaroiuGabriele LisiRoxana Valdes-RamosGabriele MascheriniRoxana-Maria AmărandiGabriele RossiRoy MoncayoGabriella EdfeldtRoy S. SmallGabriella HorváthRoyce CliffordGabriella MarféRubén AgregánGabriella MoriniRuben Lopez-BuenoGabriella Ten HaveRubén Morilla-Romero De La OsaGad SegalRubina TabassumGael MearnsRuchi RoyGaël PitonRuchika GargGael Y. RochefortRudolf LucasGaetano BergamaschiRudy J. ValentineGaetano ChinniciRuey-Yu ChenGaetano La MannaRuggiero FrancavillaGaetano MarvertiRui DuarteGaetano SantulliRui LiGaganpreet KaurRui M. Borges Dos SantosGail ReesRui PoinhosGajanan ShelkarRui VitorinoGal TsabanRuilong ShengGamal MohamedRuixue HouGang HuRu-Jeng TengGang LiRukshan MehtaGar Yee KohRulin ZhuangGary D. MillerRumiana TzonevaGary J. MyersRun-Jun YangGary MaRuojun WangGaryfallia KapravelouRupert HindsGaryfallia PeperaRusan CatarGąsowska-Bajger BeataRussell D’SouzaGaurav DhawanRussell J. MerrittGaurav JoshiRuth Adisetu PobeeGaurav V. SarodeRuth McKenzieGautam PandeyRuth T. BoachieGbemisola FadimuRuxandra StefanescuGege YanRyan LawGema Frühbeck MartínezRyan M. PaceGene-Jack WangRyan McGrathGenhong WangRyo MaekawaGeni Rodrigues SampaioRyoji FukushimaGeoff HackettRyoji YoshimuraGeoffrey MadeiraRyoma MichishitaGeoffrey UrbanskiRyota HosomiGeorg HalbeisenRyszard RafałowskiGeorge AntonogeorgosRyszard RezlerGeorge AphamisRyuji TakedaGeorge BaylissS. Udhaya KumarGeorge BraySaad HussainGeorge BriassoulisSaba BattelinoGeorge Calin DindeleganSaba TabasumGeorge DimitriadisSabata PiernoGeorge FthenakisSabeeta KapoorGeorge KarpetasSabina GaliniakGeorge NotasSabina Lachowicz-WiśniewskaGeorge P. ParaskevasSabira Mohammed JazirGeorge PaltoglouSabira SultanaGeorge PerrySabrina DemarieGeorge UmemotoSabrina EhnertGeorgia ColleluoriSabrina Gensberger-ReiglGeorgia Persephoni VoulgaridouSabrina MartinezGeorgia TrakadaSachiko KoyamaGeorgia-Eirini DeligiannidouSadaf DabeerGeorgianna TuuriSadia AfrinGeorgina Galicia-RosasSafak Guel-KleinGeorgina NakaferoSagrario Gomez-CantarinoGeorgios DimakopoulosSaida OrtolanoGeorgios PampalakisSaif KhanGeorgios TziatziosSaif Ur RehmanGeorgios TzikosSaifei LeiGerald F. WattsSakineh Kazemi NoureiniGerald H TomkinSalam IbrahimGerald KloseSalman AhmedGerald KrystalSalvador JaimeGeraldine GueronSalvador PérezGerdi TuliSalvatore Lucio CutuliGergana DeevskaSalvatore SorrentiGergő JózsaSalvatore VaccaroGeri McLeodSamanta Hernández-GarcíaGerman Dominguez VíasSamantha CatonGermán EbenspergerSamantha MaurottiGermana BanconeSamantha MoraisGerrit BegemannSameh A. AhmedGert M. KostnerSamia ElseginyGertrude ZeinstraSamira TarashiGeun-Pyo HongSamiya Al-RobaiyGhadha Ibrahim FouadSamuel CheuvrontGhassan BkailySamuel DontaGheorghe IliaSamuel Durán-AgüeroGholamreza DaryaborSamuel E. GandyGiacoma GalizziSamuele E. BurasteroGiacomo CapponSana Ben-OthmanGiacomo GaribottoSanam DolatiGiacomo RotondoSanda AndreiGiada AmodeoSandeep JagtapGianandrea PasquinelliSandeep KondakalaGiancarlo CondelloSander S. Van LeeuwenGiancarlo LucchettiSandra BarbalhoGiancarlo MichelettoSandra C. Van CalcarGianfranco AlicandroSandra Carrera-JuliáGianfranco AlpiniSandra CortesGianfranco CalogiuriSandra DonniniGianfranco NataleSandra EkströmGianfranco PelusoSandra FonsecaGianluca CatucciSandra GhelardoniGianluca FascioloSandra González-PalaciosGianluca RizzoSandra HaiderGianluca TerrinSandra HummelGianluigi MaurielloSandra Kalil BussadoriGianni CappelliSandra MooneyGiannis ArnaoutisSandra Pavičić ŽeželjGianPietro SechiSandra Roberta Gouvea FerreiraGiau VoSandra Sumalla-CanoGiia-Sheun PengSandra ZampieriGilles MithieuxSandrayee BrahmaGimeno MercedesSandrine LouisGina Marie MathewSandro Fernandes Da SilvaGina Segovia-SiapcoSandro M. HirabaraGinevra BiinoSang Heon SuhGiorgia ContaSang-Hwa LeeGiorgia MoschettiSang-Hwan DoGiorgia PallafacchinaSang-Nam KimGiorgia VaralloSangyong ChoiGiorgina Barbara PiccoliSangyong JungGiorgio BasileSaniye BiliciGiorgio BedogniSanja Klobučar MajanovićGiorgio BoganiSanjay PatelGiorgio Ivan RussoSanjeet MehariyaGiovanna BertiniSanjoy BhattacharyaGiovanna Calixto AndradeSanjoy SahaGiovanna CaparelloSantiago Navas-CarreteroGiovanna DistefanoSantonicola AntonellaGiovanna GhianiSantosh K. YadavGiovanna GiovinazzoSara Alonso-TorreGiovanna RigilloSara BecerrilGiovanna RussoSara Castro-BarqueroGiovanna TrainaSara ChiappalupiGiovanni CasellaSara Della TorreGiovanni ChiariniSara E. WuehlerGiovanni DamianiSara Fleet MichaliszynGiovanni De PergolaSara HedayatiGiovanni FarelloSara Larsson LönnGiovanni Mario PesSara MoazzenGiovanni MessinaSara NunesGiovanni PalleschiSara QuandtGiovanni PallioSara RamírezGiovanni PasseriSara Rosalía Morcuende FernándezGiovanni PeiraSara SalatinGiovanni RostiSara SalucciGiovanni SantacroceSara SaviniGiovanni SartoreSara SilaGiovanni TarantinoSarah A. JohnsonGiovanni TossettaSarah DuinGirish C. MelkaniSarah E. SaintGiuditta Fiorella SchiavanoSarah EdwardsGiulia AbateSarah J. BorengasserGiulia CarusoSarah J. Breese McCoyGiulia DonadelSarah K. G JensenGiulia GuarnieriSarah MisyakGiulia RodaSarah OddouxGiulia TestaSarah Turpin-NolanGiulia TurriSarath VijayakumarGiuliana BrunoSargis KarapetyanGiuliana MuzioSaria HassanGiuliana ScarpatiSarina KajaniGiulio ArcangeliSarit PalGiulio Francesco RomitiSaroj KhatiwadaGiulio MarchesiniSaroja VorugantiGiuseppe AnnunziataSaša FrankGiuseppe BanfiSasa MissoniGiuseppe BattagliaSascha C.A.T. VerbruggenGiuseppe CastaldoSathish ThirunavukkarasuGiuseppe DefeudisSathiyaseelan AnbazhaganGiuseppe Della PepaSathya VelmuruganGiuseppe FerrandinoSatilmis BilginGiuseppe ForteSatish GandhesiriGiuseppe GrossoSatish RainaGiuseppe LosurdoSatoru WatanabeGiuseppe ManninoSatoru YamadaGiuseppe MurdacaSatoshi KawakamiGiuseppe RizzoSatoshi NagaokaGiuseppe Russolillo FemeníasSatoshi YuguchiGiuseppe SiragoSatu K. JyväkorpiGiuseppe VisaniSaujanya KarkiGiuseppina CantarellaSavini SaraGiuseppina PalladiniSawako HibinoGiuseppina PiazzollaSchohraya SpahisGiusy Rita CaponioScott A. ReadGiusy TassoneScott ForbesGladys Oluyemisi Latunde-DadaScott T. McEwenGlayciane Costa GoisSebastian DahleGlenn P. DorsamSebastian GranicaGloria Bueno-LozanoSebastian KochGloria H. Y. LiSebastian T. BalbachGloria Jiménez-MarínSebastiano Alfio TorrisiGloria K.W. LeungSébastien GuillaumeGloria OlasoSebastien LacroixGloria Pérez-RubioSebastjan BevcGloria SalazarSee Ling LoyGloria Solano-AguilarSeeHoe NgGökçe Şeker KaratoprakSeema KumariGoldy GeorgeSehar IqbalGoncalo BarretoSe-Jae KimGonçalo SantinhaSelena E. BartlettGonzalo Solís SánchezSelene Valerino-PereaGoodarz DanaeiSeley GharaneiGopinath M. SundaramSelma CvijeticGoran GajskiSenem KamilogluGoran KrstacicSenthilkumar SankararamanGordana Kenđel JovanovićSeo Young KangGordon BarbaraSeok Shin TanGordon DrydenSeong Lim LeeGordon L. KleinSeong-Hee Maria KoGordon SmithSepalika Swarnamali Baththana MudiyanselageGoreti BotelhoSepanta HosseinpourGórnicka MagdalenaSepideh Aminzadeh-GohariGowtham K AnnarapuSepideh Zununi-vahedGraça SoveralSerafín MurilloGrace FarhatSerena AmiciGraciela Padilla-CastilloSerena Della ValleGrady NelsonSergej PirkmajerGraham HorganSergey KozlovGraham R. McGinnisSergey MaksimovGrażyna BudrynSergio Antonio TripodiGrażyna Cacak-PietrzakSergio Marques BorghiGrażyna Czaja-BulsaSergio Martinez-HervasGrażyna JanikowskaSergio Pérez-BurilloGrażyna Jarząbek-BieleckaSergio Rius-PérezGrażyna NowickaSergio VerdGrażyna Odrowaz-SypniewskaSerguei IlchenkoGreg ShearerSergueï O. FetissovGregorio Paolo MilaniSerkan YílmazGregorio PeronSeung-Geun LeeGregory C. HendersonSeungho RyuGregory ChingSeungjun LeeGregory GuthrieSevcan BakkalogluGregory J GolladaySevgi KolaylıGreta KrešićSevgül DönmezGretchen A. CasazzaSevinci PopGrethe HyldigŞeyda BostancıGrith MøllerSeyed Khosrow TayebatiGrzegorz BartoszSeyed Mohammad Taghi GharibzahediGrzegorz GajosShabam MominGrzegorz JakubiakShafiq Ur RahmanGrzegorz ZielińskiShaghayegh Baradaran GhavamiGualtiero AlvisiShahid Ali RajputGuangde JiangShahla WunderlichGuangjie ChenShailendra SinghGuangping ChenShaimaa SelimGuan-Jhong HuangShalamar D. SibleyGuanying XuShamik MajumdarGuglielmina Alessandra ChimientiShaminie AthinarayananGuglielmo CampusShan ZhengGuglielmo DurantiShanlin KeGuido ManfrediShanmuga S MahalingamGuilherme De Freitas FonsecaShannon LennonGuillaume GrzychShao-Hsuan KaoGuillaume PortierShaojun LiGuillermina CuadradoShaonong DangGuillermo CeballosSharon InmanGuillermo López LluchSharon LadymanGuillermo PetzoldSharon M. DonovanGuillermo RipollSharon R. SmithGuillermo Santos-SánchezSharzad MohseniGuiomar MasipShashwati MathurkarGül Fatma YarımShaun SabicoGuna LeeShaw-Ji ChenGunilla WieslanderShe Yu LiGunter KuhnleSheau Ching ChaiGunter WeissSheik Pran Babu Sardar PashaGuobin XiaSheila Cox SullivanGuofang ShenSheila FleischhackerGuojun WuSheila GahaganGuoxun ChenSheila M. BradyGurdeep MarwarhaSheila VeroneseGurpreet Kaur AroraSheila WeinmannGurusamy DhandapaniShelia FleischhackerGustavo Duarte PimentelSheng ZhuGustavo FernandesShengbao CaiGustavo PimentelShengshuai ShanGuy R. AdamiSheraz AliGuy RostokerSherin SaheeraGvozden RosicSherouk FoudaGwang-Woong GoShi YaoGynendra Kumar RaiShidrokh GoudarziGytė DamulevičienėShigeho TanakaGyung-Ah WieShigeki FuruyaHabiba I. AliShigeki MatsubaraHack-Lyoung KimShigeki SugawaraHae Jeong NamShigenobu ShibataHae-Rahn BaeShigeru MurakamiHai LiShigeru NakaiHai WangShih-Heng ChenHaibing ChenShih-Yi HuangHai-Hua ChuangShiming LiHaiqiu HuangShimon Ben-ShabatHaixin PengShing-Hong LiuHajime SuzukiShing-Hwa LiuHajo HaaseShingo NakahataHalil Ibrahim TanriverdiShin-Ichi KayanoHalina Cichoz-LachShin-Ichiro MiuraHalina StaniekShinsuke MizutaniHamdi ChtourouShinsuke NagamiHamidreza ArdalaniShinta NishiokaHamish CrocketShin-Tsu ChangHammad UllahShinya HasegawaHamza El HadiShiro TochitaniHan MeiShiva FaghihHan Shi Jocelyn ChewShiva Hadi EsfahaniHana FakhouryShiva HemmatiHanefí ÖzbekShivangi InamdarHa-Neui KimShixiong WangHani Ba-AwadhShizuo SakamotoHanifa BachouShofiul AzamHanna DanielewiczShoji TsujiHanna Górska-WarsewiczShoko HaraHanna SzaeferShooka MohammadiHannah H. CovertShozo YanoHannah LaneShravan GiradaHannele Yki-JarvinenShrilaxmi BagaliHanseob JeongShu WakinoHans-Georg ClassenShuen Yee LeeHans-Peter KubisShufa DuHanvedes DaovisanShuhei TsujiHany H. ArabShuhei YamadaHao ChenShuichi SuzukiHao LiShu-Jui KuoHao MaShula ShazmanHaoan ZhaoShu-Lan YehHarald EhrhardtShuling HsiehHari Prasad DevkotaShunji OshimaHaribondhu SarmaShweta Pankajkumar LavaniaHarikanth VenkannagariShwu-Huey YangHarm VelingShyam Kishor SahHarold MourasShyam MoreHarriet JohanssonShyamala ThirunavukkarasuHarriet OkronipaSibhghatulla ShaikhHarry A. QuigleySiddharth NarayananHaruki UsudaSıddika Songül YalçınHary L. RazafindralamboSiegfried WeißHaseeb ZubairSiemowit MuszyńskiHasnah HaronSigal LevyHassan BarakatSihui MaHassan RasouliSilvana AlfeiHauke ThomsenSilvana BalzanHavovi ChichgerSilvana BordinHayato TadaSilvano DragonieriHayato TerayamaSilverio RotondiHayley T. NichollsSilvia Alejandra García-GascaHazel H. OonSílvia Almeida CardosoHazuki TamadaSilvia BistiHe N. XuSilvia CanudasHeath G. GasierSilvia CapuaniHeather L. BurrowsSilvia CondeHeather Norman-BurgdolfSilvia GiannattasioHeather TickSilvia LandiHeather WilkinsSilvia LeonciniHeba AbdelrazekSilvia LiscianiHeba SahyonSilvia MarracciHebah Al UbeedSilvia Navarro-PradoHee Kyoung JooSilvia P. Tsanova-SavovaHee-Shang YounSilvia PerzolliHeeSoon LeeSílvia PinhãoHeewon JungSilvia Regina Dias Medici SaldivaHefei ZhaoSilvia RiboHeidi Vanden BrinkSílvia Rocha-RodriguesHeiko BrunsSilvia SacchiHeiko BuggerSilvia SalvatoreHeitor O. SantosSilvia SavastanoHelen McCarthySilvio BorrelliHelen VidotSime VersicHelena FelgueirasSimon CameronHelena LoureiroSimon CapewellHelena Maria André BoliniSimon FoxHelena PachónSimon Vlad LucaHelena Sandoval InsaustiSimon WelhamHélène RoumesSimona BoHelmut FrohnhofenSimona BungauHendrik GremmelsSimona CensiHendrik NolteSimona DanieleHenk Maria KoningSimona Di FrancescoHenna MuzaffarSimona GavrilasHenning FenselauSimona MirelHenning SommermeyerSimona SagonaHenriette HeinrichSimona ZaamiHenrik Max JensenSimone BattagliaHenrik OsterSimone BehrensHenry H. RuizSimone CerattoHenry J. ThompsonSimone GarzonHenry LukaskiSimone MazzuttiHenry PleassSimone PernaHenry PownallSina GalloHenry SutantoSine Roelsgaard OblingHerbert L. MeiselmanSinead N. DugganHerbert Ryan MariniSing-Chung LiHerminia Brites DiasSinghal RashiHesham El-SeediSiqi HuHideaki KawabataSiqi LiHideaki ShigaSira NanthapisalHidehiro OkuSi-Tong ChenHidekatsu YanaiSivarajan KumarasamyHideko SoneSjurdur F OlsenHidenori AkutsuSlavica KvolikHideoki FukuokaSlawomir GonkowskiHidetaka HamasakiSławomir GonkowskiHilda E. GhadiehSlawomir WybraniecHimani PuniaSławomir ZmonarskiHiroaki IoSławomira Drzymala-CzyzHiroaki KanouchiSławomira Drzymała-CzyżHiroaki SuzukiSmita S. GhareHiroe TobaSo Young KimHirohisa IzumiSobek CarolinHiroki SatoSofia AgriopoulouHiroki YokotaSofia MocoHiromi InabaSofia PereiraHiromi SatoSofia ReisHiromichi ShojiSofía Rincón-Gallardo PatiñoHiroo ImaiSohail MushtaqHiroshi IchikawaSoheb Anwar MohammedHiroshi KishimotoSoheil SobhanardakaniHiroshi SakaueSohini ChakrabortyHiroshi TamuraSok Kuan WongHirotada SuzukiSokou RozetaHiroyasu MoriSom DevHiroyuki NakamuraSon Dong-JuHiroyuki SakakibaraSonali NashineHisayo YokoyamaSonani Ravi RaghavHisayoshi KawaharaSong GaoHitoshi KagayaSoni DhruvKumarHiu Chuen LokSonia CiprianiHo Sun ShonSonia FantoneHoi Leong Xavier WongSonia GonzálezHolger SchulzeSonia Pascual-TeresaHomero MartinezSonia Rodríguez-RamírezHong JiangSonia Santander BallestinHong Sheng ChengSonja Forss-PetterHong YuSonja LacknerHongbing FanSonya MeyerHongbing SunSonya S. ShinHong-Duck KimSonya VastoHongfei ZhaoSoochong KimHongtao JinSook-Hyun ParkHongwei WangSoon Yew TangHongxiang LiuSophia LetsiouHongyi ZhouSophia LinHon-Yi ShiSophie PilleronHooi-Leng SerSoraya PazHope WeilerSoren U. NielsenHossam El-Sheikh AliSorout ShaliniHossein Tabatabaei-JafariSotero Serrate MengueHouda GharsallahSotirios KakavasHoward P. GlauertSotiris-Spyros VamvakasHrvoje KarninčićSoumojit PalHsiang-Ruei LiaoSousana PapadopoulouHsin-Chieh LanSowjanya ThatikondaHsing-Yi ChangSowmyanarayanan ThuppalHsin-Jen ChenSowndramalingam SankaralingamHsin-Tang LinŠpela KonjarHsin-Yi YangSpencer D. ProctorHsin-Yu ChenSperanza EspositoHsiu Yueh LiuSpiro M. KonstantinovHsiu-Ching ChiuSpiros VlahopoulosHsiu-Chuan ChouSpyridon KanellakisHsiu-Mei ChiangSpyridon PetropoulosHsiuying WangSpyros KontelesHsu-Heng YenSree Deepthi MuthukrishnanHua-Chen ChanSree V. ChintapalliHuachun CuiSrinath PashikantiHuaifu DengSrinidhi JayaramanHuaiyu ZhangSrinivas PittalaHuang Chung-HsiungStacey SloneHuang WeiStacy Gelhaus WendellHuawei ZengStanislav KotlyarovHubert DobrowolskiStanisław MlekoHubertus HimmerichStanley ChanHuda Al HouraniStav ShapiraHueng-Chuen FanStavros PlessasHugh GashStavroula IliaHugo De GrooteStavroula ParastatidouHugo Pliego-CortésStefan AcostaHugo Pomares-MillanStefan J. LangHui CaiStefan JoppHui ZhaoStefan PierzynowskiHui-Chen LoStefan PilzHuicui MengStefan StorcksdieckHui-Hui XiaoStefan TyskiHuijuan ZhengStefan WoelflHuili PangStefania De SantisHui-Pei HuangStefania MaiHui-Ting YangStefania MarsiliHuixian HongStefania ProiettiHui-Ying LukStefania VetranoHumaira JamshedStefanie DererHumberto Hernández SánchezStefano CacciatoreHung-Chih ChiuStefano CanosaHung-Ju LiaoStefano GovoniHung-Lung ChiangStefano LorenzettiHung-Pin TuStefano MassagliaHung-Tsung WuStefano PalermiHunter BennettStefano PiazzaHunter W. KorsmoStefano Piero Bernardo CioffiHunter WaldmanStefano PredieriHussein SabitStefano TuroloHussein SakrStefano VerdrameHwang EuejungStefanos LeontopoulosHye Chang RhimStella O’ConnellHye Won KangStella VolpeHyemin MinStepan FeduniwHyeong-Geug KimStephan KrähenbühlHyosang BaeStephan WuestHyun Joon KimStephane BourqueHyun Joong KimStéphane BurteyHyun Sook KimStéphane IsnardHyun-Jae ShinStephanie Ann FlowersI. P. KuranovaStephanie AtkinsonIain BrownleeStephanie Bader-MittermaierIain D. CroallStephanie BonnIan G. DaviesStephanie BozonetIan GriffinStephanie CurentonIbironke PopoolaStephanie Howard WilsherIbrahim BoussaadStephanie HoweIbrahim KhalifaStephanie HunterIbrahim M. SayedStephanie K. NishiI-Chien WuStephanie KurtiI-Chun TsaiStephanie SisleyIciar AstiasaranStephanie V. WrottesleyIdan GorenStephen B. SmithIdolo TedescoStephen BaileyIfeanyi OnorStephen BurnsIga RybickaStephen FallowsIgnacio BejaranoStephen InnsIgnacio Díez LópezStephen JonesIgor BuzalewiczStephen KeenanIgor DumicStephen RobinsonIgor ŁoniewskiStephen ZwolinskyIgor Piotr TurkiewiczSteve CarlanIgor ShmarakovSteve JefferyIgor TomasevicSteve LeuIgor Y. IskusnykhSteve Simpson-YapIjaz GulSteven BaumruckerIkram AliSteven M. SchwarzIl Soo MoonSteven T. LeachIla MalteseStyliani GeronikolouIlaria BortoneStyliani MinoudiIldus AhmetovSubhadeep ChakrabartiIlhami YukselSubhash KukrejaIlhem MessaoudiSubhash TripathiIllimar AltosaarSubhayan ChattopadhyayIlona GórnaSubina UpadhyayaIman ZareiSubramanian PalanisamyImmacolata Cristina NettoreSucheta TelangImtiyaz Ahmad WaniSuchira GallageIn-Ah LeeSudarshan BhattacharjeeInaki ElioSudha RajInes Bilic-ĆurčićSudhanshu ShekharInes BrandaoSudhir AggarwalInes DrenjančevićSudip BanerjeeInés Jiménez VarasSue BuckleyInes Panjkota KrbavčićSugasini DhavamaniInes SimoesSuguo HuoInês Sousa LimaSuhail AkhtarInes Vieira Da SilvaSuhas Sampat KharatInes VillanoSuh-Ching YangInga CiprovičaSu-Jin JungInga ThorsdottirSuk-Ying TsangInga WeßelsSuling HuangIngeborg BrouwerSullivan Mitchell AIngeborg KlymiukSuman ChaudharyIngibjörg GunnarsdóttirSuman ChowdhuryIngrid PrkačinSumanto HaldarIngunn BergetSumei LiuIn-Lu Amy LiuSu-Mei XiaoInmaculada Bautista CastañoSumesh ParatInsaf BerrazagaSumino YanaseIn-Sook KwunSun ChengyiIoana BalanSun Yung LyIoana CabaSung Noh HongIoana Corina BocsanSung Sam GongIoana GrigorașSung Soo KimIoana IonitaSung-Chou LiIoanna Panagiota KalafatiSunghoon ShinIoannis KarabagiasSung-Hyen LeeIoannis LiampasSunil VernekarIoav CabantchikSunmin ParkIon Alexandru BobulescuSuresh NarayanasamyIonela Ruxandra SfeatcuSuresh VeeraperumalIonut DonoiuSusan C. HuIppokratis MessaritakisSusan Fairweather-TaitIram LiaqatSusan M. SmithIrena MilaniakSusan Margaret WnukIrena MladenovaSusan Michelle GrossIrena SmagaSusan SergeantIrene AthanassakisSusan SissonIrene CaffaSusan TawiaIrene HatsuSusan TorresIrene RutiglianoSusan WhitingIreneusz KapustaSusana CardosoIrfan A. RatherSusana M. CardosoIrini ManoliSusana Rovira-LlopisIris IglesiaSusana Sánchez-FidalgoIris ScalaSusana Sangiao-AlvarellosIris TrefflichSusanna C LarssonIsaac AmoahSusanna KariluotoIsaac González-SantoyoSusanna KugelbergIsaac Yves Lopes MacêdoSusanne AufreiterIsabel AndradeSusanne RospleszczIsabel CarmoSusumu KawakamiIsabel Corrales-GutiérrezSusumu MatsukumaIsabel Escobio-PrietoSuvasini SharmaIsabel GálvezSu-Ying TsaiIsabel M. VicarioSuzanne De La MonteIsabel PrietoSuzanne FordIsabel RodriguezSuzanne SpenceIsabel Rubio-AliagaSuzette SørensenIsabel Ten-DoménechSuzy WeemsIsabel V. FigueiredoSvein Oskar FrigstadIsabella FrancoSven F. GarbadeIsabelle ChantretSven GöpelIsabelle LoganSvetlana NepocatychIsadora BeghettiSvetlana SarantsevaI-Shiang TzengSvetlana TomićIsidro Vitoria MiñanaSvetoslav TodorovIskandar IdrisSvetozár DluholuckýIsoken OlomuSvjetlana MohrmannIsolina Riaño-GalánSwati GargIsrael Villarrasa-SapiñaSyed Haris OmarIstván Adorján SzabóSyed Husain Mustafa RizviItandehui Castro QuezadaSylvère StörmannIuliana NenuSylvia CrowderIurii KobozievSylvia E. BadonIurii S. StafeevSylvia MignonIva HojsakSylvie BerthozIva Marques-LopesSylvie Hauguel-de MouzonIvan Cruz-ChamorroSylwester ŚlusarczykIvan ĆukSylwia Katarzyna KałuckaIvan Delgado EncisoSylwia MałgorzewiczIvan DimauroSylwia Onacik-GurIván Herrera-PecoSylwia PłaczkowskaIvan KreftSylwia Zakowska-BiemansIvan LandiresSzabolcs VarbiroIvan Luzardo-OcampoSzakács ZsoltIvan UherSze ChanIvana BlešićSze Yen TanIvana DjuricicSzu-Tah ChenIvana GiangriecoSzymon TomczakIvana JarakTabussam TufailIvana KmetičTack-Joong KimIvana NikolicTada-aki KudoIvana NovakTadahiro YahagiIvana Pavlinac DodigTadashi NakamuraIvana Rumora SamarinTadayoshi KagiyaIvana SamarzijaTadeusz AmbrozyIwata OzakiTadeusz DerezinskiIwona BonieckaTadeusz PawelczykIwona KonopkaTae Ha ChungIwona KowalczukTae Im KimIwona Maria ZarnowskaTae-Kang KimIwona Markiewicz-GórkaTaghi MiriIwona Piatkowska-ChmielTaiki MiyazawaIwona PuzioTakafumi AndoIwona RudkowskaTakahisa OhtaIwona TraczykTakakazu MitaniIwona Wojtasik-KalinowskaTakao KatoIwona WybrańskaTakao KimuraIza F. Peréz-RamírezTakao SaitoIzabel Cristina Rodrigues Da SilvaTakashi HimotoIzabela BolesławskaTakashi ItoIzabela Grabska-KobyleckaTakashi ObamaIzabela Szymczak-PajorTakashi UebansoIzabela ZakrockaTakayoshi AkimotoIzabella UchmanowiczTakayuki MasakiIzumi KajiTakayuki ToshimitsuJ. Alberto Montoya-AlonsoTakemichi FukasawaJ. Patrick O’ConnorTakeshi IzawaJ. S. RanTakeshi KamiyaJ. Scott LeeTakeshi UemuraJ. W. HanrahanTakeshi YamaguchiJ.A. RathmacherTakuji TanakaJ.A.P.M. De LaatTakuro TobinaJ.D. AdamsTakuto FujiiJ.T. McLaughlinTakuya SugaharaJacalyn McCombTalha Bin EmranJacek BoguckiTalwinder S. KahlonJacek PiatekTamami OdaiJacek SobockiTamás DecsiJacek TabarkiewiczTamas MarosvolgyiJacob A. SiedlikTamas SzaboJacob RaberTammy KindelJacob WilsonTan DunxianJacopo Junio Valerio BrancaTania FiaschiJacopo PruccoliTânia MartinsJacopo TalluriTânia MonizJacqueline A. PettersenTanis R FentonJacqueline C. MitchellTanja WernerJacqueline KentTanjina RahmanJacqueline M. BentelTanushree BanerjeeJacquelyn BertrandTao LanJacques BindelsTao TongJacques RigoTaotao DaiJadranka VranekovićȚarălungă Dragoș DanielJadwiga AmbroszkiewiczTarek SolimanJadwiga HamułkaTariq AbdulKarim AlalwanJadwiga ManiewskaTariq AzizJadwiga TurłoTarja KokkolaJae KimTaryn J. SmithJae Seung KangTatiana A. ZenchenkoJaeho ChungTatiana ArmeniJae-hyun KwonTatiana ChristidesJaemin LeeTatiana El-BachaJaewoo ChoiTatiana RogasevskaiaJagdish KhubchandaniTatiana Yu KuznetsovaJagoda O. SzafrańskaTatijana ZemunikJahidul IslamTatsuhiro HisatsuneJai-Eun KimTatsuo KandaJaime DevineTatsuro EgawaJaime GahcheTatsuya FujikawaJair Adriano Kopke De AguiarTatsuya KoyamaJakir HossainTatsuya MorimotoJakub KlimkiewiczTatsuya SekiguchiJakub KortasTaylor C. WallaceJakub MorzeTed WilsonJakub PiwowarskiTejas KarhadkarJalal TaneeraTelma QuintelaJames A. HendrixTeodora IvanovaJames BerkleyTeofana-Otilia Bizerea-MogaJames BuszkiewiczTeresa CardosoJames C. HurleyTeresa Da CostaJames CuffeTeresa LeszczyńskaJames DorlingTeresa M. SgaramellaJames FellTeresa PadroJames HebertTeresa PerraJames Kevin SummersTeresa W. JohnsonJames M. ChurchTereza GrimmichováJames N. TsoporisTerje Larsen TromsoJames PowersTerrence DonohueJames R. ConnorTerue KawabataJames R. HebertTeruyoshi AmagaiJames R. StubbsTeruyoshi TanakaJames X. HartmannTessa R. EnglundJameson ForsterTetsumori YamashimaJamioł-Milc DominikaTetsuo ShojiJan BilskiTetsuya HirataJan BlusztajnTetsuya ShiuchiJan CalissendorffThais Fernanda De Campos Fraga Da SilvaJan ChojnackiThaís R. SilvaJan DamoiseauxThais SteemburgoJan HellerThao PhamJan HenzelThao T.B. HoJan HošekThavaree ThilavechJan MieszkowskiTheerut LuangmonkongJan NovákTheocharis IspoglouJan P. RöerTheodora NikouJan PithaTheodora PapamitsouJan SchuchardtTheodore I. SteinmanJan TrnaTheodoros SergentanisJan WenzelTheoni KanellopoulouJan Willem Van Der KampTheresa A MikhailovJan WintrichTherese Fostervold MathisenJana BabjakováThilina U. JayawardenaJana KatuchovaThinni Nurul RochmahJane D. LaniganThirunavukkarasu SathishJane FletcherThiruventhan KarunakaranJane ZieglerThiyagarajan VaradharajanJanet FranklinThomas BaranowskiJanet LordThomas BeiklerJanet Snell-BergeonThomas BiasJanice FletcherThomas CampbellJanice SychThomas DandekarJanine L. WrightThomas G. RiemerJanna G. KoppeThomas MetaxasJanne HukkanenThomas N. MaloneyJanneke P. Van WijngaardenThomas OngJanusz KapuśniakThomas PolakJanusz KsiazykThomas SandersJanusz SolskiThomas SkurkJara Pérez-JiménezThomas WeltonJarl BøgwaldThomas WoleverJarlei FiamonciniThu H. LeJaromir AstlThuli DwebaJaromir LachmanTian JingzhiJaroslaw CzubinskyTian LiJarosław CzyżTibebeselassie Seyoum KeflieJarosław J. SakTibor ErtlJarosław KrzywańskiTiebing LiangJarosław PasekTien Sy DongJarosław WidelskiTiffany J. Ríos-FullerJasbir UpadhyayaTilakavati KarupaiahJasna KusturicaTilman KühnJasna TwynstraTim WalkerJason BrumittTim ZiegenfussJason Daniel WagganerTimothy CiesielskiJason JabbariTimothy H. MoranJason T. TzenTimothy NguyenJasper GrashuisTimothy SimeoneJatin KalitaTimotius Ivan HariyantoJaume AmengualTina M. LowreyJavad AlizargarTina MoffatJavaid NaumanTina SanghviJavier Blasco-AlonsoTina TomazicJavier Conde ArandaTinchun ChuJavier Eloy Martínez GuiraoTingtao ChenJavier FontechaTingting JuJavier Frontiñán-RubioTing-Yun LinJavier Germán Rodríguez-CarpenaTin-Yun HoJavier MarianiTish KnobfJavier ModregoTiziana CiarambinoJavier Ruiz RamírezTiziana CollettiJavier Sanz-ValeroTiziano VerriJay CaoToan Nguyen-SyJay TrivediTobias GorisJaya AseervathamTodor TodorovJayanth RamadossTomas BaležentisJayapal Reddy MallareddyTomas FiedlerJayasimha Rayalu DaddamTomás José González-LópezJaycob D. WarfelTomasz BlicharskiJayong ChungTomasz GołębiowskiJeadran Malagón-RojasTomasz GrzywaczJean ButeauTomasz HikawczukJean ButelTomasz KobielaJean DemarquoyTomasz KostkaJean H. KimTomasz LesiowJean KerverTomasz OwczarekJean Luc OlivierTomasz PodgórskiJean M KerverTomasz PoplawskiJeanett Friis RohdeTomasz ŚledzińskiJeanette M. AndradeTomasz WróbelJean-François GiguèreTomer Ziv-BaranJean-Frédéric BrunTomislav KizivatJean-Louis GueantTomiyo NakamuraJean-Marc ZinggTommaso BucciJean-Marie RobineTommaso FilippiniJean-Michel LecerfTommy Cabeza De BacaJeanne MurphyTomohiro UdagawaJeanne NerbonneTomokazu OhishiJeannette SchenkTomonori OkamuraJean-Paul MottaTomoo OkadaJean-Philippe HaymannTomotaka UgaiJean-Philippe KriegerTomoya FujieJean-Pierre ChouraquiTomozumi TakataniJean-Pierre FauvelTong Ho KangJedrzej AntosiewiczTong WangJeeser Alves De AlmeidaToni PonsJeevan PrasainToni SteerJee-Young MoonToni Torres-McGeheeJeffery HeilesonTonia VassilakouJeffrey CraigToshiaki OharaJeffrey E. CassisiToshie FujishimaJeffrey EckertToshifumi OsakaJeffrey WigleToshiharu FurukawaJelena Marković FilipovićToshihiro NomuraJelena MileševićToshikazu SuzukiJelena Osmanović BarilarToshiko TanakaJelena TošovićToshimi ChibaJelena ViderToshio NorikuraJen-Chieh TsaiToshiyuki NakaoJenette CreaneyTovit RosenzweigJenifer MonksTracy L. OliverJenni FirrmanTriantafyllos DidangelosJennifer BrayTrifan Anca-VictoritaJennifer FalbeTristan Chalvon-DemersayJennifer GjerdeTsan-Chi ChenJennifer J. McIntoshTsukasa YoshidaJennifer JordanTsukui TakayukiJennifer L. P. ProtudjerTsung-Jen WangJennifer L. PomeranzTsuyoshi ChibaJennifer MytychTsvetelina VelikovaJennifer OseTuba EsatbeyogluJennifer SalinasTuck Seng ChengJennifer SneddonTudor Lucian PopJennifer SonesTudor Sorin PopJenny HouchinsTung Yu TsuiJenny Vilchis-GilTuo HuJen-Yin ChenTurner H. SwartzJeong-Chae LeeTusneem KausarJeonghoon HaTuula Sontag-StrohmJeong-Hwa WooTuyen Van DuongJérémie NeastaTvrtko HudolinJer-Ming ChangTwan AmericaJerónimo Aragón VelaTyler J TitcombJerónimo González-BernalTzuan Ann ChenJerry PoleselTzu-Shing DengJerzy BertrandtUdaiyappan JanakiramanJerzy ChudekUday N. ShivajiJerzy JuśkiewiczUday PratapJesse C. KrakauerUjang TinggiJessica A. GriegerUlf SchottJessica Graus WooUlises De La Cruz-MossoJessica M. FerrellUlisses Moreno-CelisJessica PepeUlla UusitaloJessy EtienneUlrich DesselbergerJesus BalsindeUlrich HübnerJesús Francisco García GavilanUlrich KellerJesús OsadaUlrich MassingJesús PalomeroUlrich Severin DischingerJesús Siquier CollUlrich ZisslerJesús VioqueUlrika Von DöbelnJhen-Wei RuanUlrike LindequistJi Eun KimUlrike ReschJi Yeon KimUlrike ToepelJi Youn YooUmberto GaragiolaJiachi ChiouUmberto TarantinoJia-Ching ShiehUmesh D. WankhadeJia-Feng ChangUndine LehmannJiahao PengUri AlkanJiajia SongUroš ČakarJiale YangUrszula GuzikJian LiUrszula Krupa-KozakJian LiuUta JappeJianfeng HuangUte AlexyJiangtao DuUtoomporn SurayotJianhong ChingUtthapon IssaraJianjun WenUyory ChoeJiann-Chu ChenVaclav BuncJiann-Ruey HongVahid FarzanehJianqiang XuVal Andrew FajardoJiantao MaValentín E. Fernández-ElíasJianzhou CuiValentina BaccoliniJiaxin LingValentina BoscaroJia-Yao DoongValentina BucciarelliJia-Ying SungValentina CaputiJiayue GuoValentina De CosmiJichun ChenValentina GiorgioJie GaoValentina Lucia La RosaJie LeeValentina MeliniJie TianValentina Oana BudaJieping YangValentina PozziJihye KimValentina Rosa LaganàJill C. NewmanValentina VelleccoJim DavieValentini KonstantinidouJimi FrancisValeria PasciuJimin YangValeria PolzonettiJimmy T. EfirdValeria Rachela VillellaJin LeeValeria SogosJin ZhaoValerie CopieJina HongValérie MarcilJinchuang ZhangValerie StullJin-Chul KimValerio GiacconeJing PangValerio IzziJing WangValerio LeoniJingbo PangValério Monteiro-NetoJingjing ZhangValeriu LupuJingli XieValeriu Marin ȘurlinJingyi ChiValery FilippovJinhu GuoValery PavskyJinli WangValery V. LikhvantsevJinming HanValeryia MikalayevaJinn-Rung KuoVan Bon NguyenJin-Seng LinVanathi PerumalJirayu TanprasertsukVanda AndradeJitendra Kumar SinhaVanesa CepasJiufeng WangVanessa BortoluzziJiyoung BaeVanessa De La Cruz-GóngoraJo KatoVanessa Faria CortesJoachim SchnurbusVanessa Liévin-Le MoalJoan Aureli Cadefau SurrocaVanessa PorriniJoan Francesc Fondevila GascónVanessa Stadlbauer-KöllnerJoan LiVânia Marilande CeccattoJoan MontanerVaromyalin TipmaneeJoan QuilesVarsha SheteJoana Abou-RizkVartika TomarJoana CostaVasanth KumarJoana GasparVasile DrugJoana GonçalvesVasileios PapapanagiotouJoana Pereira De Carvalho FerreiraVasilevskaya EkaterinaJoanna Bartkowiak-WieczorekVasiliki LeventakouJoanna BoniorVassilis PaschalisJoanna BrysVatcharapan UmpaichitraJoanna Gdula-ArgasińskaVeera C. S. R. ChittepuJoanna Gromadzka-OstrowskaVeera KainulainenJoanna GrzelczykVelda GonzalezJoanna KamińskaVelia CassanoJoanna KocotVenkatakrishnan RengarajanJoanna KowalkowskaVenkataraghavan RamamoorthyJoanna LeszczyńskaVered Kaufman-ShriquiJoanna Maria SuliburskaVerena M. H. TanJoanna Myszkowska-RyciakVerginica SchröderJoanna OswiecimskaVeronica Alexandra AntipovaJoanna PieczyńskaVerónica CornejoJoanna Raszeja-WyszomirskaVeronica RodillaJoanna Seliga-SiweckaVerónica SeguraJoanna SikoraVeronika SomozaJoanna SuliburskaVéronique FontaineJoanna TeichertVesa Cosmin MihaiJoanna WezgowiecVessela BalabanovaJoanna WietrzykVibha SinghalJoanna WojtkiewiczVibhav GautamJoanna ZarzynskaVic Ben-EzraJoAnne ArcandVicente Calixto Zanón-MorenoJoanne BrookeVicente Pallarés-CarrataláJo-Anne PuddephattVicente PerezJoão Antônio Leal De MirandaVictor BanerjeeJoão Batista Ferreira‐JuniorVíctor García-GonzálezJoão LeitãoVictor GaultJoão LimaVictor Manuel Mendoza-NuñezJoão Paulo Saraiva MoraisVictor PulgarJoaquim Calvo-LermaVictor R. GordeukJoaquim CarrerasVictor TaleonJoaquim Manuel Trigo MarquêsVictoria AbrilJoaquim PinheiroVictoria L. StevensJoaquín González-RódenasVictoria Valls-BellésJocelyn E. RemmertVida JuozaitienėJocelyn R. LebowViera KaraffovaJocelyne R. BenatarVijay KumarJochen SchneiderVijay LyallJodi Dunmeyer StookeyVijay Rakesh Reddy SanikommuJody DushayVijaya Anand ArumugamJoe MillwardVijayakumar MavanjiJoel C. GeerlingVikrant RaiJöerg HeerenVilelMine CarayanniJoeri J. PenVilhelmova-Ilieva NeliJoeri Jan PenVillinger KarolineJoffrey ZollVilma KaškonienėJohan De Vogel-Van Den BoschVilma KriaucionieneJohan MolenbroekVilnis DzerveJohan P. WoelberVinal MenonJohannes J RaskerVincent FontaineJohannes SchulzeVincent PonsJohannes SimonsVincent V. BekkerJohn A. BatsisVincent Van BuulJohn A. CaldwellVincent Y-H ChengJohn A. RathmacherVincenza CofiniJohn AggasVincenzo CastelloneJohn B. LasekanVincenzo De LeoJohn Bertram VincentVincenzo GianturcoJohn C. PetersVincenzo MarcotrigianoJohn DotisVincenzo MollaceJohn DubeVincenzo QuagliarielloJohn H. FosterVinicius CruzatJohn J. GuersVinicius Silva CastroJohn JonesVinita BharatJohn KanakakisVinny NegiJohn KearneyViola Maria PopovJohn M. PerryVirgilio Lima GómezJohn M. ThompsonVirginia BoccardiJohn O. WarnerVirginia TancrediJohn WhitehallVirginia UhleyJolanta ChmielowiecVishal ChavdaJolanta Krzysztoń-RussjanVishma Pratap SurJolanta LewkoVishnu KhanalJolanta Lis-KuberkaVisnja KaticJolanta Nawrocka-RutkowskaVitaly PostoevJolanta RzymowskaVito TerlizziJolene ZhengVitor Andre Silva VidalJolita RadušienėVitor RodriguesJon A. VanderhoofVittoria BorgonettiJon HanfVittoria CammisottoJon IrazustaVittorio CalabreseJon LundbergVivek KumarJonas ViškelisVivek PecheJonatan MirandaVivek PuriJonatas Rafael De OliveiraViviana BarraJonathan D. KarpViviana Loria KohenJonathan KershawVivien ChavanelleJonathan M. ScottVladana GrabezJong-Hwa KimVladareanu RaduJong-Koo KimVladiana TuriJong-Won KimVladimir A. ShvartzJonhatan SilvaVladimir DyakonovJonnathan Guadalupe Santillán-BenítezVladimir PisarevJoo Young HuhVladimir PorochJoohyung LeeVladimir SerezhenkovJooMan ParkVlaho BrailoJoon W. ShimVlasta DostalovaJoonhong ParkVlastimil KuldaJordi EspadalerWahajuddin WahajuddinJordi Espadaler MazoWai Phyo AungJordi SerraWalburga DieterichJörg H. StehleWaleska C DornasJörg RadermacherWalied Mohamed AlarifJorg Van LoosdregtWalter CurrentiJorge Arturo Santos LópezWan Abd Al-Qadar Imad Bin Wan MohtarJorge CervantesWan MananJorge H. VargasWan NamkungJorge M. CavigliaWan ShenJorge Moreno-FernandezWan-Chun ChiuJorge R. Soliz-RuedaWang LeiJoris R. DelangheWang QiJorja CollinsWang ZhengJosca M. SchoonejansWang Zheng-XinJosé AgudeloWaseem MushtaqJose Alvarez-SuarezWatanabe KoichiJose AntonioWei Hsum YapJose Antonio De Paz FernándezWei YingJosé Antonio Fernández-LópezWei ZhangJosé Antonio García-ErceWei ZhuJosé Antonio García-SantosWeilai DongJose Antonio Hurtado-SánchezWei-Min ChenJosé Antonio Picó-MonllorWeiqing MinJosé Aparecido Da SilvaWeiran ShanJose AppolinarioWeiwei CuiJosé AssumpçãoWenchao YangJosé Augusto SimõesWendy HuntJose C. E. SerranoWendy J. O’BrienJose CanasWenfei ZhuJosé ChiesWenhan YangJosé Claudio Fonseca MoreiraWen-Huang PengJosé Cleiton Sousa Dos SantosWenhui XieJose CorderoWenJie LiJosé Daniel Jiménez-GarcíaWenjie ZhouJosé Enrique De La Rubia OrtíWen-Jun ShenJosé Francisco López-GilWenjun ZhangJosé G. Fernadez-BolanosWenqing XuJose Geraldo MillWen-Tzu WuJosé Ignacio Baile AyensaWen-Wei ChangJosé J Jiménez-MoleónWenxin SongJosé JusticiaWenxin TongJose L. CarballoWenyen JuanJose L. Gómez-UrquizaWhittington JenniferJose L. Quiles MoralesWiesław CubałaJose Laparra LlopisWilbert P. VermeijJose Lara GallegosWilhelm MistiaenJosé Luis MansurWill K. ReevesJosé Luis Martín-ContyWilliam B. GrantJosé Luis Pérez-CastrillónWilliam BarbeauJosé Luis Romero-BejarWilliam BlanerJose Luis UbagoWilliam FraserJosé Luiz De Brito AlvesWilliam JacotJose M. GamonalesWilliam ManJosé M. JiménezWilliam RassmanJose M. MirandaWilliam S. BlanerJosé Manuel López GarciaWilliam WoodwardJose Manuel MoralesWilliam Y. RaynorJosé Manuel Moreno VillaresWilma Maria Coelho AraújoJosé María HuertaWim CalameJosé Miguel González-ClementeWim J.E. TissingJosé Miguel Martínez-SanzWinfried L. NeuhuberJose Moisés LaparraWioletta PawlukowskaJose MoranWioletta Szczurek-WasilewiczJose PuzoWioletta Zukiewicz-SobczakJosé UberosWiramon RungratanawanichJose-Benito Rosales ChavezWirginia TomczakJosef FlammerWisit CheungpasitpornJosefa Gonzalez-SantosWissem MnifJosely KouryWitold KędzierskiJosep JulveWi-Young SoJosep Maria Ramon TorrellWladimir Bocca Vieira De Rezende PintoJoseph K. PellegrinoWładysław LasońJoseph L. RobertsWojciech JelskiJoseph L. SáenzWojciech KochJoshua BarzilayWojciech ŁuczajJoshua C. DoloffWojciech PiekoszewskiJoshua CameronWojciech SkrobotJoshua M. KaplanWolfgang BernhardJoshua W. MillerWolfgang BuchallaJosip DelmisWolfgang H. HartlJosip VrdoljakWolfgang HerrmannJosipa BukicWonsuk AnJosipa RadićWoochun JunJosje SchoufourWoojin JunJoydeep ChakrabortyWoon-Man KungJožef ŠimenkoWouter KokJózsef DeliWouter R. P. H. Van De WorpJuan Armendariz-BorundaWubin XieJuan BlasiXi ChenJuan Camilo Henao-RojasXi YuJuan Carlos MontejoXiang FeiJuan Diego Ramos-PichardoXiang LiJuan J. CabréXiangli ZhaoJuan José DíazXiangpeng KongJuan José Hernández MoranteXiangpeng LiaoJuan José MorenoXiangsheng FuJuan Jose SalineroXiangyu RenJuan L. HernandezXiangyu ZhangJuan LlopisXiangzhu ZhuJuan Luis Bonilla PalomasXiao TanJuan M. PericàsXiaodan HuangJuan M. Serrador PeiróXiaodong LiJuan Manuel Guzmán-FloresXiaofeng LiuJuan Manuel Muriel-SánchezXiaohong ShuJuan Miguel Martínez-GalianoXiao-hua WangJuan Mozas-MorenoXiaoju HuJuan Torres-MachoXiaolei TangJuana Maria Morillas RuizXiaoliang WangJudit BajorXiaomeng YouJudith A. BetoXiaoping PuJudith A. SmithXiaoqin WangJudith FlanaganXiaorong FuJudith MihalyXiaowen HuJudith SligoXiaoya ShangJudith StorchXiaoyan WangJuei-Tang ChengXiaoying WangJuergen DreweXiaoyu ZhangJuergen SchrezenmeirXiaoyun LiJuha HulmiXiaozhen LiuJuha TaavelaXiaxia DiJulia M. PotterXibi FangJulia Wojciechowska-SolisXie BingJulian FriebelXin ChenJuliana ChenXin LinJuliane CalvezXin LiuJuliano GaravagliaXin TongJulie C. AbayomiXin YeJulie HessXin ZhangJulie JonesXinchen WuJulie LaniganXing ZhangJulie Miller JonesXingao ZhangJulien Saint-PolXinghua WangJulio Alvarez PittiXingshun QiJulio Calleja-GonzalezXingyi JiangJulio OchoaXin-Hua XiaoJulio Plaza DíazXinli LiJuliusz PrzyslawskiXin-Tang LinJuliusz PrzysławskiXinxu YuanJumin ParkXiuyun WuJumpei SaitoXiyuan LuJun Hui JeongXóchitl TrujilloJun KobayashiXu ChenJun MiyataXu Dong ZhangJun Shi LaiXue Feng HuJun WatanabeXuehua XuJunfeng MaXuequn PangJung Eun KimXueyan FuJung Hwan OhXun SongJung-Chul ParkYa’akov HenkinJung-Heun HaYael LebenthalJung-suk SungYafeng LiJungwon MinYajun ChenJunho KimYalda Rahbar SaadatJunichi IwataYa-Li HuangJunichi MatsumotoYamixa DelgadoJunji TakayaYan BornéJunling GuoYan LamJunne-Ming SungYan ZhaoJunrui ChengYana CenJunzi WuYana SandlersJuraci A. CesarYang CaoJuraj PayerYang HeJurandir F. ComarYang LiJürgen GermannYang-Ching ChenJürgen VormannYanghoon JungJuristo FonolláYangtian YiJurriaan MesYanhui HanJustin DiAngeloYanjie ZhangJustin P. Van BeusecumYankho KaimilaJustine BoldYanna TelesJustyna BrasunYanna ZhuJustyna Brzezicha-CirockaYao SongJustyna GodosYaozhou(Sophie) ZhuJustyna Opydo-SzymaczekYara M. MichelacciJustyna PalecznyYari LongobuccoJustyna Podgórska-BednarzYaşar KaradumanJustyna Rosicka-KaczmarekYasmin JahanJustyna SteckoYasuka MatsunagaJustyna StrycharzYasuki SekiguchiJustyna ZińczukYasumasa IkedaJyh-Fei LiaoYasutada AkibaJyothi F. NagajyothiYasutaka ShigemuraJyotirmoy DasYasuyuki IshikawaK. GulyaYasuyuki NagasawaK. M. Faridul HasanYaujiunn LeeKacie HoYazeed BarghouthyKah Hui WongYe PengKa-hei Kenneth LoYen Chen TungKai Ling KongYen Po WangKai WangYen-Po ChenKaihui LuYen-Tze LiuKailash SinghYen-Tzu WuKai-Lee WangYen-Wenn LiuKaisa HiippalaYeonsoo KimKai-Wen ChengYeung Joon ChoiKai-Wen HsuYftach GepnerKalathil K SureshkumarYi Chun ChenKali MakedouYi DaiKalina DuszkaYi LuanKalliopi Anna PouliaYibo QiuKalliopi KaratziYi-Chen HsiehKalu Kapuge Asanka SanjeewaYi-Chia HuangKameron ModingYi-Chin LinKamil KarolczakYi-Ching YangKamila Czepczor-BernatYi-Chiung HsuKamila GoderskaYifan BaoKamila Pokorska-NiewiadaYi-Hung LiaoKamilla StachYi-Jen LiaoKamiński MikołajYijing ZhouKandeepan KarthigesuYijun PanKandi SridharYin-Ching ChanKanshi MinamitaniYing JinKara Ann TinkerYing LiuKaranovic DanijelaYing QingKarel AllegaertYing YangKarel D. CapekYing ZhouKaren BannertYingjun LiuKaren Chapman-NovakofskiYingnan JiaKaren KalanetraYing-Si LaiKaren LindsayYingting CaoKaren Russell-RandallYishai LevyKaren S. ByrdYi-Sub KwakKaren TanYitang SunKaren WebbYi-Yuan LinKarin B. MichelsYlenia SpissuKarin MagnussenYlva Tiblom EhrssonKarl Uwe PetersenYohann WittrantKarna RamachandraiahYoichi MatsuoKarolina A. Wojtunik-KuleszaYoichi SutohKarolina ChilickaYoichiro OdaKarolina JakubczykYoko HirataKarolina KotYoko UchiyamaKarolina Krupa-KotaraYoko YokoyamaKarolina LeopoldYolanda Terán-FigueroaKarolina OsowieckaYolande EsquirolKarolina Szewczyk-GolecYong JinKarolina TkaczYonghe MaKarthick RajanYonghong MengKarthik H. ShankarYongping BaoKarthikeyan VenkatachalamYongqiang ChenKasandra BélangerYoo Kyoung ParkKatarina SebekovaYoon Kwang LeeKatarina SmilkovYoona KimKatarina VukojevicYoshida HisakoKatarzyna BanachYoshifumi KasugaKatarzyna BergmannYoshiharu ShimomuraKatarzyna BurekYoshihiko FurutaKatarzyna CyprykYoshihiro FukumotoKatarzyna CzyżYoshihiro IkuraKatarzyna GrocholewiczYoshihiro KamadaKatarzyna HojanYoshikazu UchidaKatarzyna KaczyńskaYoshiki KoriyamaKatarzyna Kajak-SiemaszkoYoshinori KatakuraKatarzyna Kiec-KononowiczYoshinori MikamiKatarzyna KirszYoshio OguraKatarzyna KorybalskaYoshitaka FukuzawaKatarzyna Kosinska-KaczynskaYou WuKatarzyna KowalskaYouenn LohéacKatarzyna LachowiczYou-Hsiang ChuKatarzyna ŁubiechYouling XiongKatarzyna Mleczko-SaneckaYoung ChoiKatarzyna NabrdalikYoung Sub KwonKatarzyna Neffe-SkocińskaYoung Whan ChoiKatarzyna NeubauerYoungjoo SohnKatarzyna PrzybylowiczYoung-Keol ChoKatarzyna RolfYoung-Min ParkKatarzyna SkrypnikYoung-Sang KimKatarzyna StaniewskaYousef NaserzadehKatarzyna ŚwiąderYoussef RomanKatarzyna SzamotulskaYousuke TaokaKatarzyna SzkudelskaYu KoyamaKatarzyna Winsz-SzczotkaYu MengKatarzyna Wroblewska-SeniukYu XiaoKatarzyna ZawadaYu. S. SidorovaKate DentonYuan LiKate LawrenceYuan-Ti LeeKaterina GiouriYuanyuan ShiKaterina KotzampassiYu-Chen YangKaterina P. SkenderiYu-Chieh ChenKatharina C. Quack LötscherYufei ShiKatharina C. WirnitzerYugo ShibagakiKatharina DiehlYuhei OtobeKatharina RumpYuh-Jyh LinKatharina WirnitzerYuh‐Yih LinKatherine AppletonYuichiro KomaKatherine ChettaYuichiro YamashiroKatherine E. VestYuji NagatomoKatherine M. HammittYuji ShimizuKatherine OttoliniYuji TajiriKatherine P. AdamsYu-Jung ChengKathleen CarterYuki EnokiKathleen DavisYukinori YabutaKathleen KevanyYukio MaruyamaKathleen RichardsonYu-Kuo ChenKathleen V. AxenYulia Yu StroylovaKathrin OhlaYuliang SunKathryn CoakleyYulin RenKathryn F. MedlerYuling XieKathryn HartYu-lyu YehKathryn HoppertonYu-Mei HsuehKathryn M JandaYumie TakataKathryn M. LeifheitYumin DaiKathryn T. KnechtYun ShiKathryn Y. BurgeYun ZhaoKathy TaylorYun ZhuKátia FernandesYung-Chieh LinKatie Lynn SummersYung-Hsiang TsaiKatie WynneYunkoo KangKatja KröllerYunmei WangKatja ParschatYunyun GongKatja ZmitekYurii B. ShvetsovKatrin Annika Kopf-BolanzYusheng LiangKatrina BrudzynskiYusof KamisahKatrina HutchinsonYusuke MurataKatsuhiko TakahashiYusuke OhsakiKatsuhiro ItoYusuke UgataKatsumi IizukaYutang WangKatsunori NakamuraYutong DongKavita SharmaYu-Tse WuKavitha PenugondaYuya NakanoKawaguchi NanakoYuyin ZhouKayo TanakaYvan VandenplasKazue IshitsukaYves MenezoKazuhiko KotaniYvonne SingerKazuhiro NishiyamaZ. Elizabeth FloydKazuhiro P. IzawaZachary S. ClaytonKazuhisa MatsunagaZafer CeylanKazuki YasudaZahra AshkavandKazushige OshitaZahra FarahnakKazuto TajiriZainab T. Al-SharifyKazuya TateishiZakaria TazartKazuyuki FurutaŻaneta Kimber-TrojnarKe K. ZhangZbigniew AdamiakKedra WallaceZbigniew GarncarekKee-Lung ChangZbigniew KamockiKegan Romelle JonesZdenek SumnikKei NakajimaZdeněk ZadákKeigi FujiwaraZeenat MirzaKeiichi KoshinakaZeki Atıl BulutKeiko KabasawaZeljko SundovKeiko MaedaZeno StangaKeisuke KuwaharaZeynep Tacer CabaKeita KaiZezhong TianKeith MartinZhan ShuKeith RochfortZhang JihuiKelemu KibretZhang JingKelly E. JohnsonZhaoli DaiKelly MulderZheng Zachory WeiKelly S. SantangeloZhenglin GuKelsey A. SchmidtZhenglin YangKen OngZhengxiang HuangKengo IshiharaZhengyun WuKenichi GotoZhengzhong XuKenichi SakuraiZhenni ZhuKen-Ichiro KonishiZhenzhen JiaKen-Ichiro MinatoZhenzhen QinKenji SanadaZhenzhen ZhangKenneth G. GerowZheqing ZhangKenneth L. SeldeenZhi ChaiKenneth SmithZhicheng CuiKensei TaguchiZhiguo ZhangKensuke KumeZhijia TangKentaro JujoZhipeng CaoKentaro KamiyaZhipeng TaoKentaro KawabeZhiyong ZouKeren DopeltZhonghou WangKerstin KempfZhongkang YangKervin EvansZhongwei LiuKeshav Raj PaudelZhousheng XiaoKeshore BidaseeZhuqiu JinKevin HackshawZifang ZhaoKevin KantonoZijian ZhangKevin M. CumminsZijuan LiuKevin MakiZikai HaoKevin MillerŽivoslav TešićKevin ZwetslootZobia NoreenKhaled HamdenZoe AshleyKhaled TrabelsiZohair Al-AmeenKhalisanni KhalidZohar Barnett-ItzhakiKhawaja Muhammad Imran BashirZoltan H NemethKhursheed ShiekhZongkui WangKhurshid AhmadZongzhi YinKhushdeep BandeshZoubir DjeradaKhushwant Singh BhullarZsofia VerzarKhyati GirdharZubair KabirKi-Bae HongZubair KhanKiessoun KonatéZubair MalikKijin KimZufang WuKi-Jong RheeZuhair NattoKim A. WilliamsZulfitri Azuan Mat DaudKim B. PedersenZumin ShiKim F. MichaelsenZuohui ZhangKim M. Van VlietZuriaga EstefaníaKim StoteZuyi (Jacky) HuangKim-Anne LêZuzanna RzepkaKimberley WangZvonimir ŠatalićKimberly A. KewZvonko RumboldtKimberly M. JeckelZyanya Reyes-CastilloKimberly R. MiddletonZygmunt Warzecha


